# From Natural Sources to Synthetic Derivatives: The Allyl Motif as a Powerful Tool for Fragment-Based Design in Cancer Treatment

**DOI:** 10.1021/acs.jmedchem.2c01406

**Published:** 2023-03-01

**Authors:** Nora Astrain-Redin, Carmen Sanmartin, Arun K. Sharma, Daniel Plano

**Affiliations:** † Department of Pharmaceutical Technology and Chemistry, University of Navarra, E-31008 Pamplona, Spain; ‡ Department of Pharmacology, Penn State Cancer Institute, CH72, Penn State College of Medicine, 500 University Drive, Hershey, Pennsylvania 17033, United States

## Abstract

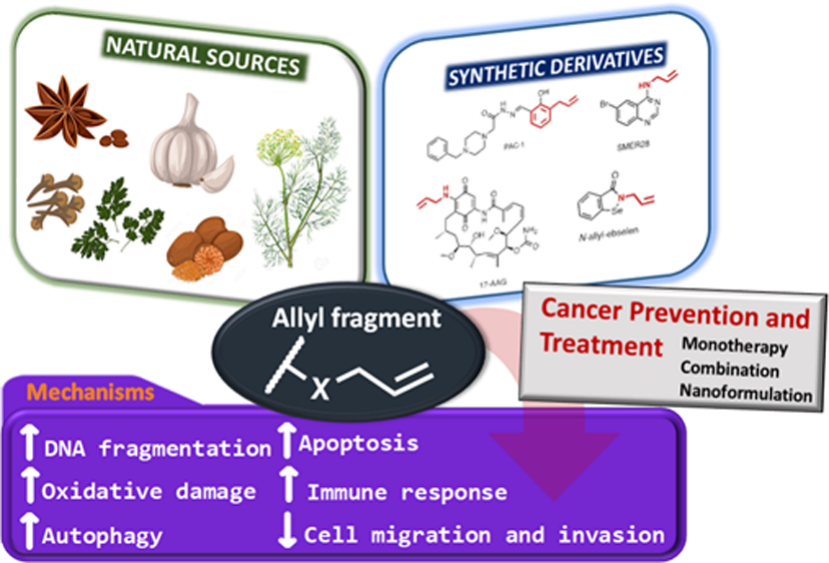

Since the beginning of history, natural products have been an abundant source of bioactive molecules for the treatment of different diseases, including cancer. Many allyl derivatives, which have shown anticancer activity both *in vitro* and *in vivo* in a large number of cancers, are bioactive molecules found in garlic, cinnamon, nutmeg, or mustard. In addition, synthetic products containing allyl fragments have been developed showing potent anticancer properties. Of particular note is the allyl derivative 17-AAG, which has been evaluated in Phase I and Phase II/III clinical trials for the treatment of multiple myeloma, metastatic melanoma, renal cancer, and breast cancer. In this Perspective, we compile extensive literature evidence with descriptions and discussions of the most recent advances in different natural and synthetic allyl derivatives that could generate cancer drug candidates in the near future.

## 1 Introduction

Cancer is one of the main causes of death worldwide, accounting for nearly 10 million deaths in 2020.^1^ Over time, the cure rate of patients has increased due to improved early diagnosis and more personalized treatments. Among these treatments are radiation therapy, surgery, immunotherapy, endocrine therapy, gene therapy, and chemotherapy, the latter being the most widely used either as monotherapy or in combination with other treatments. However, resistance to chemotherapy in aggressive cancers has increased over time, which—together with the adverse effects chemotherapy causes—has led to the need for the development of new anticancer agents.^2^

Strategies for the development of new anticancer drugs have evolved over the years from the use of molecules based on natural sources (NSs) and drug repositioning to targeted therapies. Plants have been an unlimited source of new bioactive molecules that have allowed the development of anticancer drugs such as paclitaxel, irinotecan, and vincristine.^3^ Likewise, studies of structure–activity relationships (SARs) have led to the identification of the natural molecule fragments responsible for their anticancer activity and their introduction into synthetic molecules, thus improving their activity.

**Figure 1:**
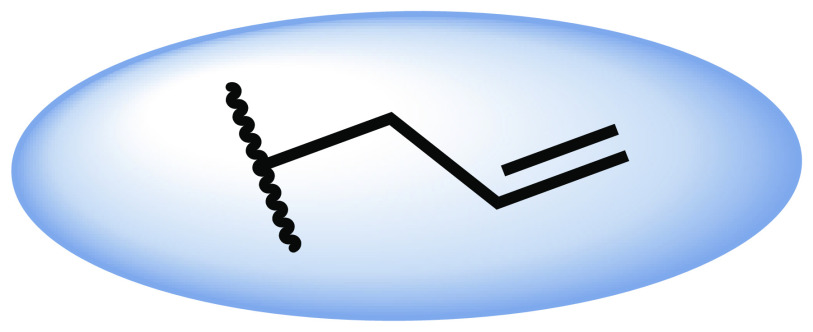
Structure of the allyl group.

In addition, several reports have shown that compounds derived from NSs, such as garlic, cinnamon, nutmeg, and mustard, possess pharmacological properties, including antitumoral activity. One thing that all of the above-mentioned natural sources have in common is that they contain bioactive molecules with allylic chains in their structures. The allyl group is formed by a methylene bridge (−CH_2_−) attached to a vinyl group (−CH=CH_2_), as depicted in Figure 1. When an allyl moiety contains a good leaving group, it readily generates an allylic cation. This cation is stabilized by mesomerism and is an excellent electrophile. Many natural compounds can liberate allylic alcohols.^4^ To date, no review has been published that brings together allylic derivatives based on natural products and their anticancer activity, as those that have been published have dealt with allylic compounds separately according to their sources, such as garlic or alkylbenzene derivatives. Therefore, this Perspective encompasses extensive literature evidence, from the past 6 years, with descriptions and discussions of the most recent advances in natural and synthetic allylic molecules and their relevance as anticancer drugs.

## 2 Natural Allylic Compounds and Cancer Therapy

Since the beginning of history, most ancient civilizations have used herbal remedies for the treatment of health issues. In the 18th century, the development of botany and organic chemistry laid the foundations for finding active substances in plants, which would later be turned into drugs. Currently, classical plant-derived drugs, such as digoxin, atropine, and ergotamine, are used in daily life.

In the search for new cancer treatments, molecules derived from NSs have always offered a relevant development pathway. It is estimated that, between 1981 and 2019, 25% of new anticancer drugs approved by regulatory agencies were related to natural products.^5^ A prominent example is paclitaxel, a chemotherapy drug that is produced from the bark of the Pacific yew, *Taxus brevifolia*.^6^ In 1979, Horwitz published the first scientific article highlighting the antimicrotubule capacity of Taxol,^7^ and in 1992 it was approved by the FDA for the treatment of recurrent advanced breast and ovarian carcinomas.^8^ Other well-known chemotherapy drugs from NSs include irinotecan (derived from *Camptotheca acuminata*), vincristine (derived from *Catharanthus roseus*), and etoposide (derived from *Podophyllum notatum*).

### 2.1 Garlic Derivatives

Garlic (*Allium sativum L.*) has been widely studied due to its pharmacological benefits which include antimicrobial, antiarrhythmic, antithrombotic, antitumoral, hypoglycemic, and hypolipidemic properties.^9^ However, the study of garlic components as antitumoral drugs has been of significant interest.^10−13^ In 1957, Carworth Farms White (CFW) Swiss mice were inoculated with sarcoma 180 ascites tumor cells that were previously incubated with a reaction mixture of the garlic enzyme alliinase and *S*-allyl-l-cysteine sulfoxide (**1**) as substrate. After 6 months, no tumor growth had occurred in the mice and the animals remained alive.^14^ This experiment was the first evidence that garlic contained bioactive molecules with antitumoral activity.

**Figure 2:**
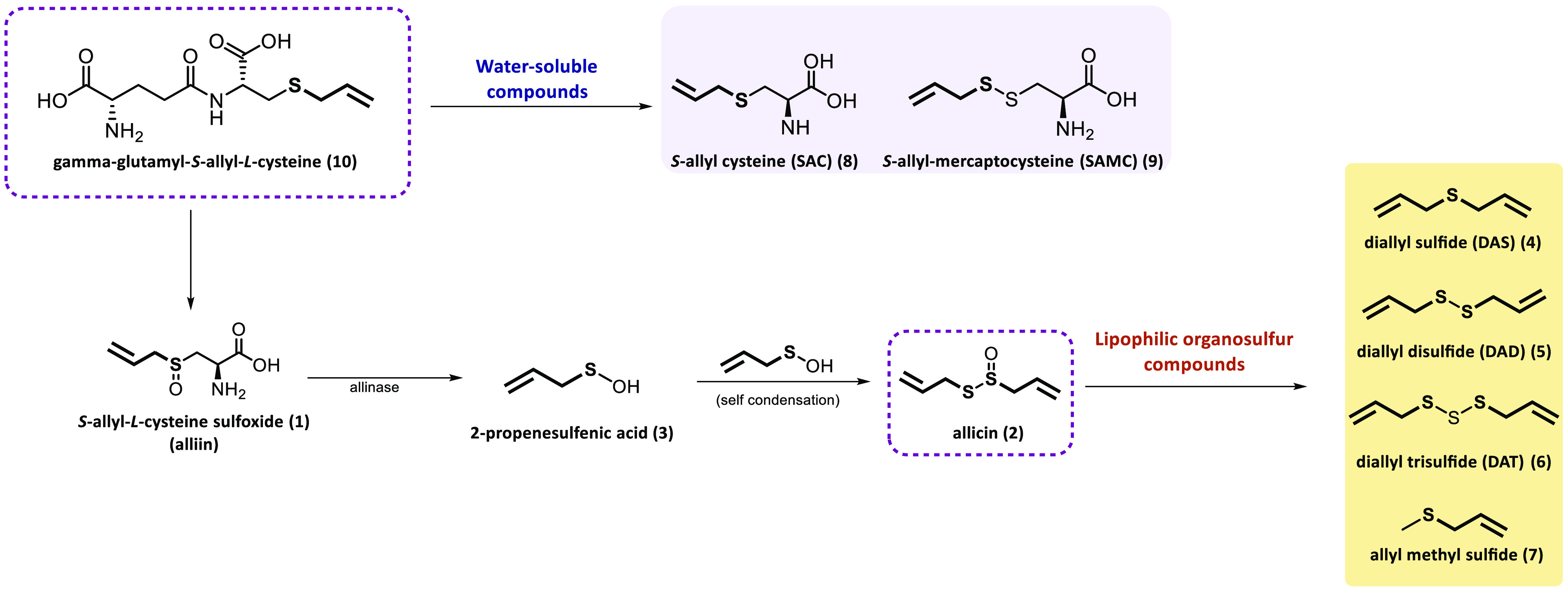
Garlic-derived compounds, with the thioallyl group highlighted in bold.

Garlic contains a wide variety of biologically active compounds, but in this Perspective, we will focus on the thioallylic molecules with antitumoral activity (Figure 2). One of these thioallylic molecules, allicin (**2**), formed by the condensation of two molecules of 2-propenesulfenic acid (**3**), forms a range of lipophilic organosulfur compounds, including diallyl sulfide (DAS, **4**), diallyl disulfide (DAD, **5**), diallyl trisulfide (DAT, **6**), and allyl methyl sulfide (**7**). Garlic also contains water-soluble allyl amino acid derivatives, including *S*-allyl-cysteine (SAC, **8**) and *S*-allyl-mercaptocysteine (SAMC, **9**), formed from γ-glutamyl-*S*-allyl-l-cysteine (**10**) after long-term fermentation of crushed garlic.^13^ All of these molecules have demonstrated activity against a multitude of human cancers through different mechanisms that include inhibition of cell proliferation and tumor growth, modulation of enzyme activities, free radical scavenging, inhibition of mutagenesis, induction of DNA damage, and cell cycle arrest.^15,16^Table 1 summarizes the antitumor activity of the different allyl derivatives present in garlic.

**Table 1:** Modes of Action of Garlic-Derived Natural Thioallyl Compounds in Cancer

Type of cancer	Mode of action	Refs
***S*-Allyl-cysteine (SAC, 8)**		
Lung (HCC827 and NCI-H1975)	Increase of oxidative damage in lipids	(18)
	Apoptosis	
	Decrease of Nrf2 and NF-kB expressions	
Lung (A549)	Reduction of PD-L1 and HIF-1α expressions	(19)
	Decrease in cell growth and proliferation	
	Apoptosis by enhanced nuclear condensation and increased percent caspase-3 activity	
Breast (MCF-7)	Decrease in cell viability through a reduction in the level of MPST-3 and sulfur sulfate	(21)
	Late apoptosis (2245 μM)	
Breast (MDA-MB-231)	Decrease in type I collagen adhesion and MMP2	(20)
	Increase of E-cadherin	

**Nonivamide-*S*-Allyl-cysteine Ester**		
Breast (MCF-7)	Increase of ROS generation and decrease of GSH level	(22)
	Decrease of Bcl/Bax ratio and mitochondrial-mediated apoptosis	
	Cell arrest in G1/S phase followed by DNA damage	
	Increase of p53 expression	
	IC_50_ value 66 μM	

**NSAIDs-*S*-Allyl-cysteine Derivatives**		
Colorectal (SW480 and CHO-K1)	Cytotoxic activity (IC_50_ values 0.131–0.183 mM)	(23)

***S*-Allyl-mercaptocysteine + Docetaxel**		
Hormone refractory prostate (PC3, DU145, and 22Rv1)	Apoptosis	(25)
	Cells arrest in G2/M phase	
	Suppression of Bcl-2 expression and increase of E-cadherin	

***S*-Allyl-mercaptocysteine + Rapamycin**		
Colon (HCT-116 *in vitro* and xenograft model in BALB/c nude mice)	p53-dependent apoptosis with an increased ratio of Bax/Bcl-2	(26)
	Inhibition of autophagy (↑LC3-II autophagy marker)	
	Inhibition of Akt phosphorylation	
	Enhance of Nrf2 pathway and down-regulation of p62	

**Allicin (**2**)**		
Gastric (miscellaneous)	Arrest cell cycle at G2/M phase	(35)
	Endoplasmic reticulum stress	
	Mitochondria-mediated apoptosis	
	Death receptor pathway	
Gastric (HGC27 and AGS)	Suppression of cell viability	(36)
	Apoptosis	
	Inhibition of cell migration and invasion through up-regulation of miR-383-5p and inhibition of ERBB4/PI3K/Akt pathway	
Colon (HCT-116)	Apoptosis by suppression of STAT3 signaling activation	(31)
	Enhancement of Nrf2 pathway	(32)
	Apoptosis with a decrease in Bcl-2 levels, increase in Bax levels and increase in cytochrome *c* release from mitochondria	

**Diallyl Sulfide (DAS, **4**)**		
Hepatocellular (HepG2 and Huh7 *in vitro* and xenograft model of HepG2 cells in BALB/c nude mice)	Apoptosis by the activation of caspase-3 and by Bax/Bcl-2 pathway	(38)
	Inhibition of ER-α36 rapid estrogen signaling	
Breast (MCF-10A)	Decrease of DNA stand breaks and lipid peroxidation	(39,41)
Prostatic (BPH-induced rats)	Suppression of testosterone level via reduction of IL-6	(43)
	Reduction on androgenic receptor expression	
	Reduction in prostatic weight by reduction of TGF-β1, IGF, and ERK	

**DAS (**4**) + Paclitaxel**		
Skin (in rats)	Apoptosis by reduction of Bcl2 and increase of p53 protein	(42)

**Diallyl Disulfide (DAD, **5**)**		
Esophageal squamous (ECA 109)	Arrest cell cycle at G2/M phase by activation of p53/p21 pathway	(48)
	Apoptosis by activating caspase-3, up-regulating Bax/Bcl-2 balance, and suppressing MEK-ERK pathway	
Prostatic (PC-3)	Caspase-3-mediated apoptosis and up-regulated Bax/Bcl-2 ratio	(49)
Leukemia (HL-60)	Inhibition of proliferation, migration, and invasion	(52)
	Decrease of cofilin 1 expression by down-regulating Rac1-ROCK1-LIMK1 signaling pathway	
Leukemia (HL-60 *in vitro* and xenograft model in Kunming species mice)	Arrest cell cycle at G0/G1 phase	(54)
	Decrease of J-1, cofilin 1, RhoGDP dissociation inhibitor 2, calreticulin and PCNA	
	Induction of cell differentiation	
Gastric (MGC803)	Inhibition of TGF-β1/Rac1 signaling pathway	(56)
Lung (A549)	Suppression of canonical Wnt signaling pathway	(57)
	Reversion of fibronectin-induced epithelial mesenchymal	
Osteosarcoma (MG-63)	Caspase-3 mediated apoptosis and up-regulated Bax/Bcl-2 ratio Inhibition of autophagy	(58)
	Arrest cell cycle at G2/M cycle	
	Inhibition of PI3K/Akt/mTOR signaling pathway	
Colon (SW480 *in vitro* and xenograft model in BALB/c nude mice)	Reduction on cell migration and invasion by suppressing the phosphorylation of ADF/cofilin via down-regulation of LIMK1	(59)
Breast (MDA-MB-231)	Decrease of the expression of CCL2/MCP-1	(66)

**DAD (**5**) Nanoformulation**		
Breast (MD-MB-231)	Apoptosis	(61)

**DAD (**5**) + Leflunomide**		
Hepatocelullar (in rats)	Up-regulation of Mfn2 expression	(63)
	Antimetastatic activity through up-regulating the expression of Timp-3 and decreasing hepatic MMP9 content	

**Diallyl Trisulfide (DAT, **6**)**		
Breast (MDA-MB-231 and HS 578T *in vitro* and xenograft model of MDA-MB-231 cells in BALB/c nude mice)	Suppression of breast cancer metastasis	(70)
	Reduction of Trx-1 nuclear translocation from cytoplasm	
	Decrease of NF-kB and MMO2-9 in primary tumor and lung tissue	
Breast (MCF-7 and SUM159)	Apoptosis	(73)
	Suppression of breast CSCs through inhibiting Wnt/β-catenin pathway activation	
Breast (MDA-MB-231)	Inhibition of HIF-1α	(80)
	Inhibition of L1CAM, VEGF-A, and EMT-related proteins	(81)
	Inhibition of α-secretases expression (ADAM10 and ADAM17)	
Colon (SW480 and DLD-1)	Inhibition of Wnt/β-catenin pathway	(75)
Sarcoma (SW928)	Apoptosis	(83)
	G2/M cell cycle arrest	
	Increase of intracellular ROS	
Gastric (BGC823 *in vitro* and xenograft model in BALB/c nude mice)	Apoptosis through the attenuation of Nrf2/Akt and activation of p38/JNK	(88)
	G2/M cell cycle arrest (cyclin A2 and B1 accumulation)	
Gastric (BGC823)	Inhibition of sulfiredoxin	(86)
	Decrease of ROS levels	
Gastric (AGS)	Apoptosis	(87)
	G2/M cell cycle arrest	
	ROS-dependent activation of AMPK pathway	
Thyroid (8580C)	Mitochondrial apoptosis	(89)
	G2/M cell cycle arrest	

**DAT (**6**) + Doxorubicin**		
Breast (Ehrlich solid carcinoma (ECS)-bearing mice)	Suppression of Notch signaling proteins (Notch 1, JAG 1, and HES 1)	(82)
	Attenuation of tumor inflammation (NFκB, TNF-α, IL-6, IL-1β) and proliferation (decrease of cyclin D1, Ki67)	
	Apoptosis via caspase-3 and p53	

**Figure 3:**
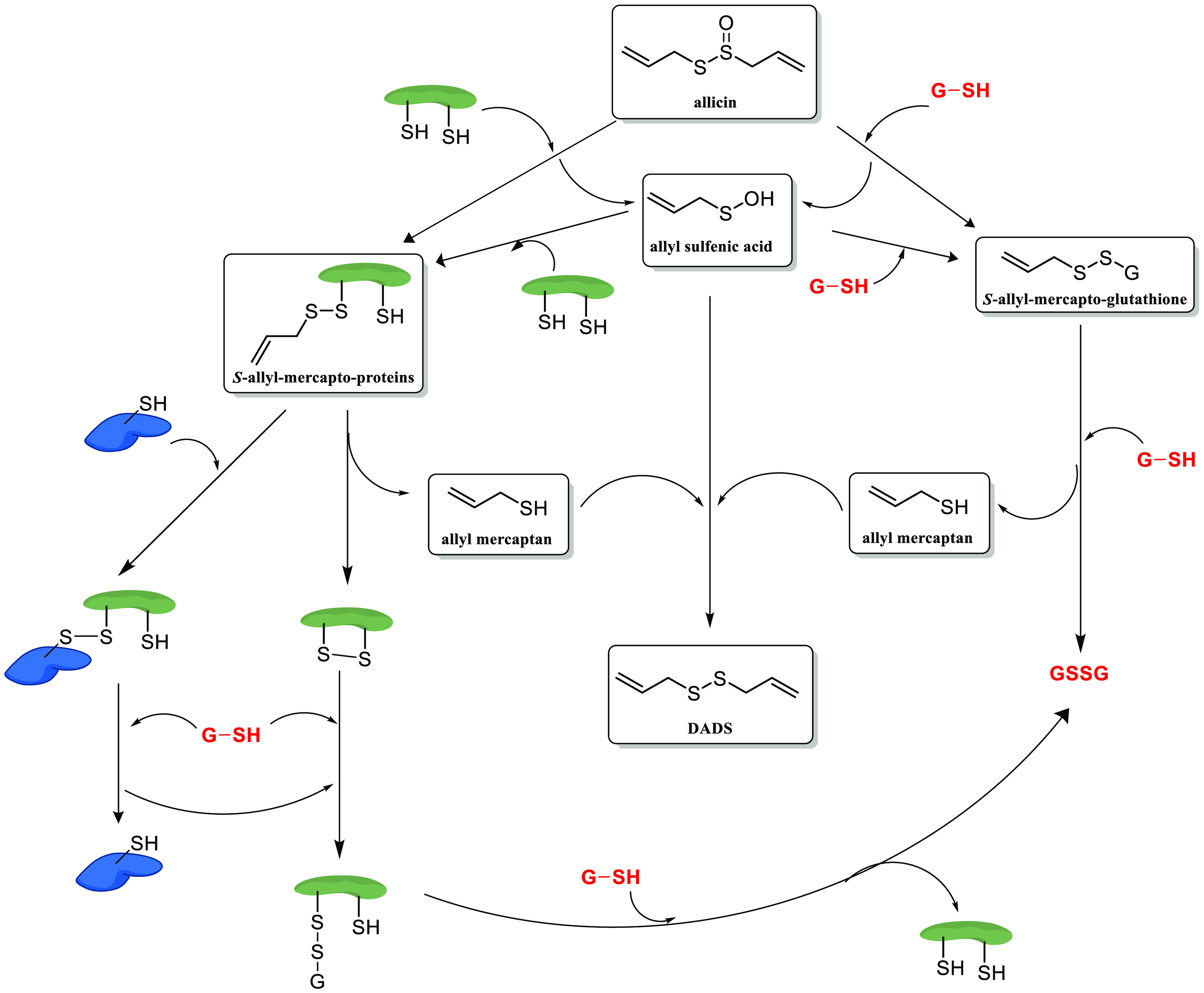
Redox chemistry of allicin: interactions with proteins containing thiol groups such as GSH or cysteine. Modified from Borlinghaus et al.^17^

To date, different mechanisms of action for garlic-derived allyl compounds in cancer have been unveiled. However, it should be noted that, in many of these, the allyl chain appears to be a mere observer in many cellular processes that these sulfur molecules may undergo, such as interaction with redox enzymes. An example is the redox chemistry of allicin (**2**). Allicin is able to interact with cellular thiols such as glutathione (GSH) or cysteine-containing proteins (Figure 3). These reactions might yield *S*-allyl-mercapto proteins and allyl-sulfenic acid, which can again interact with proteins by forming disulfide bonds, with the subsequent elimination of allyl-mercaptan. The latter can interact with another molecule of allyl-mercaptan or allyl-sulfenic acid to form DAD (Figure 3). In all of these reactions, the allylic moiety appears to act as a mere spectator since the main actor is the sulfur atom.^17^

The antitumoral activity of SAC (**8**) was studied in lung cancer cells, and it was observed that the compound not only significantly reduced cell growth and proliferation but also induced apoptosis and oxidative damage.^18^ Additionally, the therapeutic potential of SAC as a potent immune checkpoint inhibitor capable of reducing the expression of programmed death ligand 1 (PD-L1) and hypoxia-inducible factor 1 (HIF-1) in A549 lung cancer cells was also determined, which was supported by *in silico* analysis.^19^ On the other hand, in SAC-treated breast cancer MCF-7 cells, a concentration- and time-dependent decrease in cell viability was observed, along with the induction of late apoptosis. In breast cancer metastasis, SAC was able to decrease type I collagen adhesion and matrix metalloproteinase 2 (MMP2) activity, inhibiting cell mobility and migration.^20^ The significant decrease in mercaptopyruvate sulfurtransferase (MPST) and sulfane sulfur levels was one explanation of the promising effects of SAC on the deterioration of the MCF-7 cells’ condition.^21^

**Figure 4:**
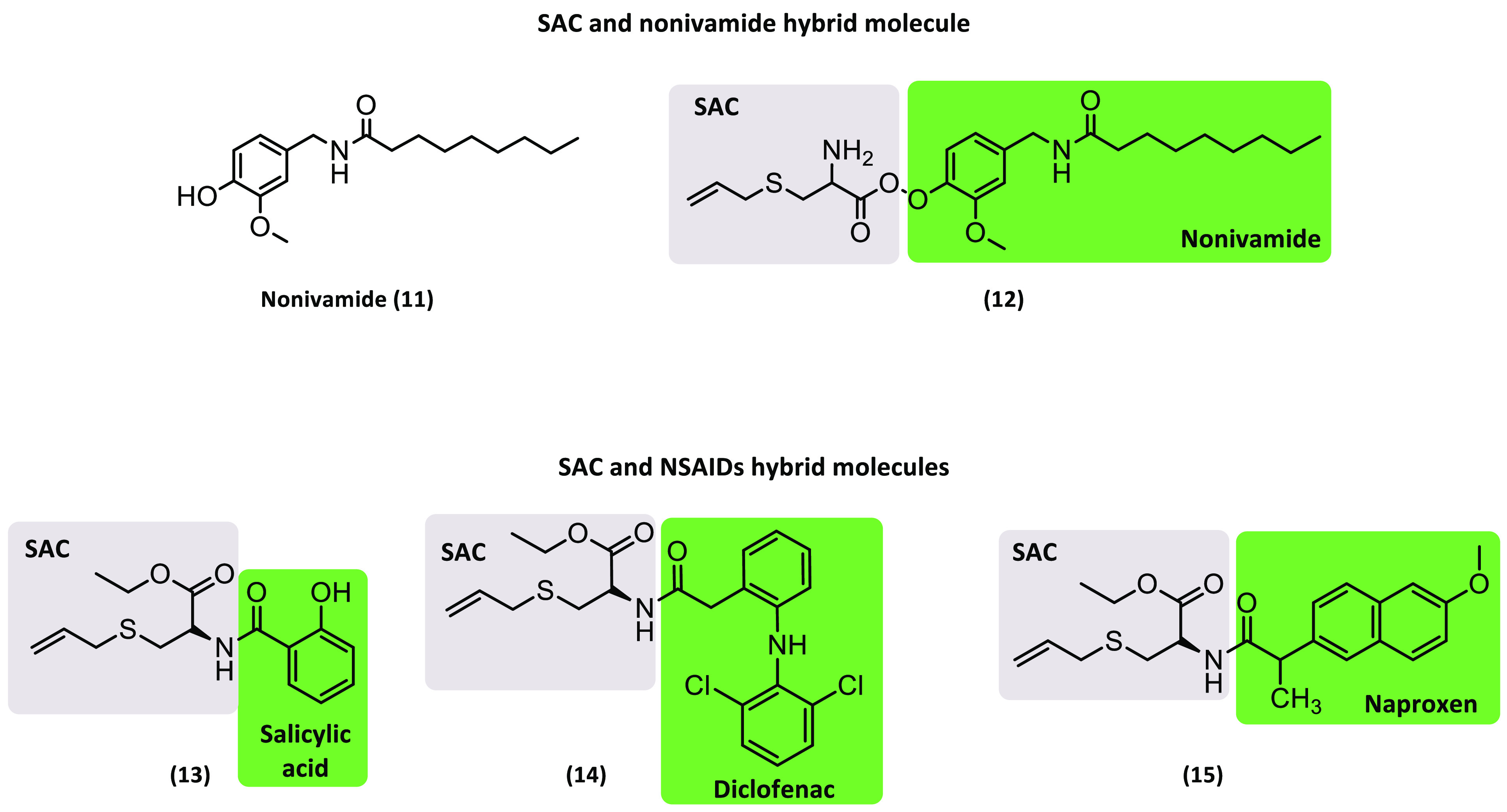
Chemical structures of hybrid derivatives from SAC.

In a search for new anticancer molecules, molecule **12** containing nonivamide (**11**), a less pungent analog of capsaicin and SAC (**8**), was developed (Figure 4). Molecular docking studies and dynamics simulation analysis suggested the potential for a stable interaction and favorable binding of the hybrid molecule **12** with therapeutic target proteins that included the human estrogen receptor α (ER-α), tumor protein negative regulator mouse double minute 2 (MDM2), B-cell lymphoma 2 (Bcl-2), and cyclin-dependent kinase 2 (CDK2) to treat cancer.^22^ These molecular docking studies suggested that **12** could interact with ERα at a site similar to that of the antagonist tamoxifen. Similarly, MDM2 plays an important role in cancer, as it is a negative regulator of the nuclear transcription factor p53. This nuclear transcription factor, in response to cellular stress, triggers transcriptional activation of the effector p21, leading to cell cycle arrest and apoptosis. Thus, up-regulation of its negative regulator MDM2 in tumor cells disables p53, enhancing cancer progression. Bcl-2 is an anti-apoptotic protein that is highly expressed in cancer cells and promotes their survival. The molecular docking study suggested that the newly synthesized molecule could act as a Bcl-2 inhibitor. CDK2 is a cell cycle regulator, and its inhibitors are considered to offer a novel strategy for cancer treatment. However, molecular docking and dynamic simulation studies are theoretical approaches that need to be confirmed by *in vitro* studies. Therefore, the anticancer activities of **12** and nonivamide (**11**) were tested against the MCF-7 breast cancer cell line. Hybrid **12** was able to decrease the viability of MCF-7 cancer cells with an IC_50_ value of 66 μM, while previous studies had shown an IC_50_ value of 2245 μM against MCF-7 cells for **11**. In addition, **12** increased reactive oxygen species (ROS) generation, arrested cells in the G1/S phase, altered mitochondrial membrane potential, and initiated DNA fragmentation. Finally, it increased p53 expression and decreased the Bcl/Bax ratio.^22^ Other SAC-derived hybrid compounds **13**–**15** were obtained by combining this fragment with non-steroidal anti-inflammatory drugs (NSAIDs), resulting in promising scaffolds for the treatment of colorectal cancer (Figure 4). The IC_50_ values for these hybrids at 24 and 48 h against colon adenocarcinoma SW480 cell line were between 0.131 and 0.183 mM, and selectivity indexes, calculated as the ratio of IC_50_ values in non-malignant CHO-K1 cells versus SW480 cells, were higher than 1 after 48 h of treatment.^23^ In the previous study and in this one, the fragment design strategy was used to develop new derivatives. This approach consists of bringing together in the same molecule two fragments that are independently active, through a weak bond. In these cases, the OH group of the nonivamide (**11**) and NSAIDs is used to form an ester bond or an amide bond with the other fragment that is incorporated into the other part of the molecule. The ester bond is a weak bond that in the body can be broken by the action of esterase enzymes to release both active moieties. The aim of this type of molecule is to obtain a more potent compound by bringing together active fragments. However, the hybrid molecules of SAC and NSAIDs have not yielded the expected results, since the IC_50_ values are not optimal for further studies of their anticancer activity against the colon adenocarcinoma SW480 cell line.

**Figure 5:**
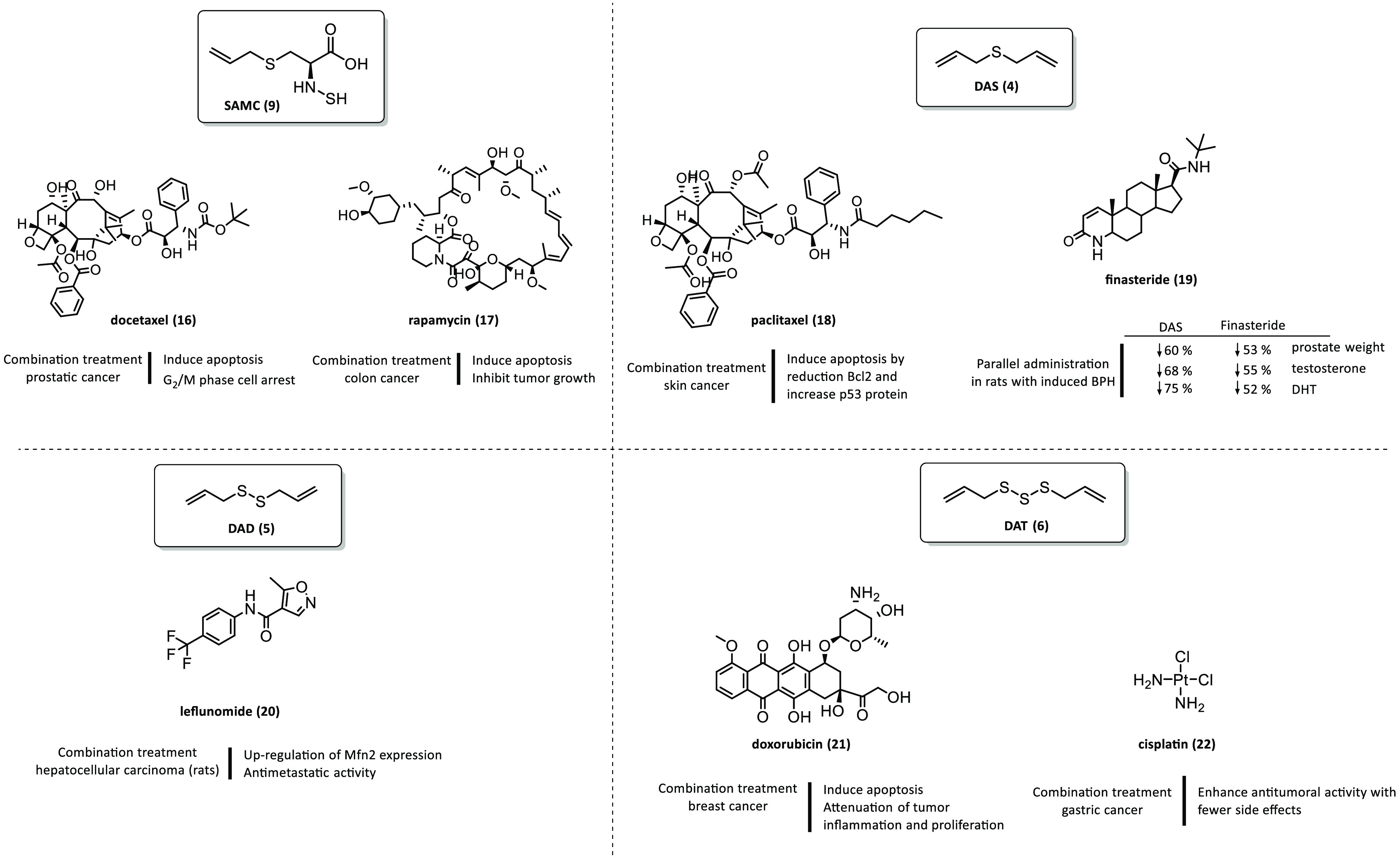
Different combination treatments with garlic-derived compounds.

The other water-soluble allyl amino acid derivative, SAMC (**9**, Figure 2), has shown anticancer activity through down-regulating Bcl-2 protein, which causes tumor cell apoptosis by a process involving activation of the mitogen-activated protein kinase (MAPK) pathway and mitochondrial cytochrome *c* release.^24^ Moreover, **9** can inhibit tumor cell proliferation by inducing histone acetylation and inhibiting microtubule polymerization. It induces E-cadherin to suppress tumor cell migration. On the other hand, the synergic administration of **9** with docetaxel (**16**) has shown apoptosis induction and G2/M phase arrest against prostatic cancer cells.^25^ In another study, **9** was shown to significantly enhance the ability of rapamycin (**17**) to induce colon cancer cell apoptosis and inhibit tumor growth in xenograft nude mice through the autophagy/p62/Nuclear factor erythroid 2-related factor 2 (Nrf2) pathway.^26^Figure 5 shows different combination treatments with garlic-derived compounds.

Allicin (**2**), which is the most abundant and the most biologically active garlic component, has shown anticancer activity *in vitro* against a range of tumor types, including breast,^27^ gastric,^28,29^ leukemia,^30^ colon,^31−33^ and renal cancer cell lines.^34^ Recent studies have suggested that **2** may exert a chemotherapeutic effect on gastric cancer cell lines by inhibiting the growth of cancer cells, arresting the cell cycle at the G2/M phase, inducing endoplasmic reticulum (ER) stress, and inducing mitochondria-mediated apoptosis, which includes the caspase-dependent/-independent and death receptor pathways.^35^ However, the main death mechanism shown by **2** is apoptosis followed by parthanatos and autophagy. The apoptotic potential of **2**, at a dose of 10 μg/mL, has been attributed to its ability to modulate a specific microRNA (miRNA), miR-383-5p, which has been demonstrated to play a role in cell proliferation, apoptosis, and differentiation, especially in gastric carcinoma.^36^ Additionally, **2** can reduce phosphorylated signal transducer and activator of transcription 3 (STAT3) to inhibit the STAT3 pathway, as well as activate Nrf2 and induce its translocation to the nucleus in colon cancer cells.^31,32^ Although the large majority of studies report that caspase-mediated apoptosis is its main mechanism of cell death, when the concentration of **2** was increased to 20 μg/mL, caspase-3 activation and cleavage of PARP were not observed, but caspase-independent apoptosis-inducing factor (AIF) was released from mitochondria.^37^ Therefore, apoptosis induced by **2** may be a concentration-dependent mechanism; however, more studies are needed to clarify this aspect of its biochemical pharmacology.

DAS (**4**), a fat-soluble compound present in garlic, has demonstrated anticancer activity against hepatocellular,^38^ breast,^39−41^ skin,^42^ and prostate^43^ carcinomas. *In vivo* studies carried out in nude mice injected with human hepatocellular carcinoma (HCC) HepG2 cells and treated with **4** revealed that it inhibited the growth and clonogenicity of HepG2 and Huh7 HCC cells. It also induced apoptosis mediated by the activation of caspase-3 with an increase of bax and a down-regulation of Bcl-2 expression. The expression levels of ER-α36 and epidermal growth factor receptor (EGFR) were also analyzed, and it was found that ER-α36 signaling is involved in the inhibition of HCC cell growth induced by **4** both *in vitro* and *in vivo*.^38^ Regarding breast cancer, **4** has been shown to effectively inhibit tumor cell proliferation, control cell cycle transitions, decrease lipid peroxidation, and attenuate DNA strand breaks.^39,41^ Combination therapy with **4** and paclitaxel (**18**) in rats with induced skin cancer not only effectively reduced Bcl-2 protein expression and increased p53 gene expression but also restored skin architecture.^42^

In another study, rats with induced benign prostatic hyperplasia (BPH) were treated either with **4** (50 mg/kg, p.o.) or with finasteride (**19**) (5 mg/kg, p.o). Finasteride (**19**) is used to treat men with BPH since it makes symptoms less severe and reduces the chance that prostate surgery will be needed. Finasteride (**19**) blocks the action of 5α-reductase, which is an intracellular enzyme that converts the androgen testosterone into 5 α-dihydrotestosterone (DHT). DHT plays a role in the development and enlargement of the prostate gland. Therefore, treatment with **19** decreases the levels of DHT, which decreases prostate size. Experimental studies in Wistar rats demonstrated that prostate weight was markedly reduced by 53% with treatment with **19** and by 60% with **4**. Additionally, serum testosterone and DHT were reduced by 55% and 52% with **19** and by 68% and 75% with **4**, respectively, in concordance with decreased protein expression of androgen receptor (AR) and prostate-specific antigen (PSA). Both **19** and **4** have also demonstrated an anti-inflammatory effect evidenced by decreased protein expression in CD4+ T-cells and reduced release of associated inflammatory cytokines. Concomitant application of **19** and **4** exhibited marked down-regulation of insulin-like growth factor-1 (IGF-1), transforming growth factor-β1 (TGF-β1), and phosphorylated extracellular signal-regulated kinase (ERK1/2) signaling. Taking all of the above into account, there is a potential therapeutic approach for **4** as a dietary preventive agent against BPH.^43^

Another thioallyl-derived compound present in garlic is DAD (**5**), which contains a disulfide functional group. A large number of studies have supported its activity as an anticancer agent, highlighting its inhibition of cancer cell migration and invasion.^44−47^ In human esophageal squamous cell carcinoma, **5** was found to reduce the number of cells in the G1 phase and to increase those in the G2/M phase, in concomitance with activation of the p53/p21 pathway.^48^ In addition to regulating cell-cycle arrest, **5** induced apoptosis through activation of caspase-3 and release of cytochrome *c* from mitochondria in PC-3 cells.^49^ Other mechanisms of action of **5** have been studied, such as the link between **5** and cofilin 1 in leukemia. Cofilin is an actin-binding protein that can depolymerize actin filaments and regulate the cytoskeleton. There are two mammalian cofilin gene subtypes, cofilin 1 and cofilin 2. Cofilin 1 has been shown to be highly expressed in several cancer types and is associated with proliferation, migration, invasion, differentiation, metastasis, and poor prognosis.^50,51^ Therefore, cofilin 1 silencing by **5** leads to inhibition of proliferation and induced differentiation of leukemia HL-60 cells.^52^ In addition, **5** has been shown to inhibit the growth of tumor tissue in immunodeficient mice injected with HL-60 cells.^53^ As well as being able to inhibit the proliferation of, migration of, and invasion by leukemia cancer cells, **5** can arrest cells at the G0/G1 stage at low doses (8 μM). On the other hand, analysis of how **5** induced differentiation found four up-regulated proteins, including galactin-10, plectin 1, AUF1, and electron transfer flavoprotein α-subunit, and 14 down-regulated proteins, including DJ-1, cofilin 1, RhoGDP dissociation inhibitor 2 (RhoGDI2), calreticulin (CTR), and proliferating cell nuclear antigen (PCNA).^54^ All of the above suggest that the effects of **5** on leukemia cells may be due to the modulation of multiple targets. It is important to note that many target proteins may have cysteines in the active site that are key to their biological activity. These allylic sulfur compounds, such as **5**, present the capacity to interact with the key thiol groups of these biological proteins through disulfide bond formation, leading to inactivation. However, there are no studies analyzing biological targets where garlic-derived compounds are shown to exert their action through sulfenylation. On the contrary, a series of 2-sulfonylpyridines have been identified that react selectively with biological thiols of adenosine deaminase via nucleophilic aromatic substitution. They react selectively with a cysteine distal to the active site, attenuating enzymatic activity and inhibiting lymphocytic cell proliferation.^55^ Additionally, different fragments, including double bonds activated to be attacked by a nucleophile, also showed their ability to exert biological activity through sulfenylation with cysteine residues. Thus, this study illustrates how the development of thiol-group-modifying molecules can be a breakthrough in the area of medicinal chemistry. Not only are these molecules worthy of further study, we also believe that compounds containing certain allylic fragments could exert antitumoral activity through this pathway. Hence, this structural feature should be considered in the design of novel allylic derivatives.

The effect of **5** on gastric cancer cell growth has been also reported. It can block transforming TGF-β1/Rac1 signaling, which may be responsible for the suppression of epithelial–mesenchymal transition (EMT), invasion, and tumor growth.^56^ Likewise, **5** can reverse the EMT induced by fibronectin, a known inducer of invasion and metastasis, via suppression of Wnt signaling in non-small-cell lung cancer.^57^ Moreover, the ability of **5** to inhibit cell viability in MG-63 osteosarcoma cells in a dose- and time-dependent manner has been demonstrated. The study revealed cell cycle arrest in the G2/M phase, as well as induction of autophagy and apoptosis through inhibition of the PI3K/Akt/mTOR signaling pathway.^58^

In colon cancer cell lines, LIM kinase 1 (LIMK1) has emerged as a potential therapeutic target since its overexpression in this type of cancer is associated with increased migration and invasion of colon cancer cells. A study was performed in which **5** inhibited cell migration and invasion by suppressing the phosphorylation of actin-depolymerization factor (ADF)/cofilin in colon SW480 cells. The inhibition of phosphorylation by **5** was effected via down-regulation of LIMK1, a result that may suggest LIMK1 as a potential target molecule in this type of cancer.^59^

Recently, new therapeutic targets for the treatment of cancer, including the receptor for advanced glycation end products (RAGE) for the treatment of triple-negative breast cancer (TNBC),^60^ have emerged. The aim of these studies is to achieve targeted treatment according to the type of cancer the patient is suffering from. For example, lipid nanoparticle formulation of DAD with a RAGE antibody loaded on its surface was tested in TNBC cells. This targeted nanoformulation achieved a significant increase in the cytotoxic effect compared to **5**-loaded nanoparticles without RAGE on their surface.^61^ On the other hand, DAD was also studied as a synergistic treatment with leflunomide (**20**) against HCC. Leflunomide (**20**) is an FDA-approved drug for rheumatoid arthritis, but it has been found to activate mitofusin (Mfn) expression, which is down-regulated in HCC.^62^ The combined treatment with **5** and **20***in vivo* showed a more potent effect than treatments with each drug alone. The treatment shifted mitochondrial dynamics toward mitochondrial fusion by up-regulating the expression of Mfn2, and it exhibited antimetastatic activity by up-regulating the expression of metallopeptidase inhibitor 3 (Timp-3) and decreasing hepatic matrix metallopeptidase 9 (MMP9) content.^63^ Another potential therapeutic target for cancer is the C–C motif chemokine ligand 2 (CCL2), which is overexpressed in cancer cells. Additionally, high CCL2 levels are associated with more aggressive malignancies, a high probability of metastasis, and a poor prognosis in a wide variety of cancers.^64,65^ In this context, **5** was able to significantly decrease the expression of CCL2/MCP-1 in TNFα-induced TNBC cells.^66^ The CCL2 chemokine is characterized by two adjacent cysteines that can be sulfenylated by **5**. However, there have not been any studies conducted that analyze in depth this possible mechanism of action.

DAT (**6**), another bioactive compound derived from garlic, contains three sulfur atoms. Several studies have reported its promising anticancer activity in various carcinomas. One of the most studied has been breast cancer, since it is the most common cancer among women. The three major subtypes are HER2+, estrogen/progesterone positive-receptor, and TNBC.^67^ The thioredoxin (Trx) system, which plays a key role in breast cancer metastasis, could be a therapeutic target. The Trx system is an efficient antioxidant system that protects cancer cells from oxidative damage.^68,69^ Garlic-derived **6** has been reported to inhibit Trx reductase and the expression of Trx-1 in breast cancer cells.^70^ Specifically, **6** reduced Trx-1 nuclear translocation from the cytoplasm, with the consequent reduction of Trx-1 formation. *In vivo* results revealed that **6** administration significantly suppressed spontaneous and experimental metastasis in a xenograft model of MDA-MB-231 cells in BALB/c nude mice. DAT (**6**) was given daily by oral administration at doses of 25 and 50 mg/kg from day 3, and all mice were sacrificed 23 days following tumor injection.^70^ As breast cancer is very heterogeneous both morphologically and molecularly, new therapeutic targets are emerging. Apart from the two mentioned above, CCL2 and the Trx system, **6** has been evaluated against other potential targets in breast cancer, including the canonical Wnt/β-catenin signal pathway, histone deacetylase enzymes, and α-secretases. The canonical Wnt/β-catenin signal pathway is crucial for maintaining cancer stem cell (CSC) characteristics.^71,72^ It has been reported that **6** not only could effectively inhibit the viability of breast CSCs, as evidenced by reduced tumorsphere formation, decreased expression of breast CSC markers, inhibition of proliferation, and induction of apoptosis, but also could reduce the activity of the Wnt/β-catenin pathway.^73^ Similarly, another study supported the ability of **6** to modulate the Wnt/β-catenin pathway in human bronchial epithelial sphere-forming cells exposed to chronic tobacco smoke (the main cause of lung cancer).^74^ DAT (**6**) also suppressed the activity of the Wnt/β-catenin pathway in colorectal CSCs.^75^ Thus, it seems that inhibition of the Wnt/β-catenin pathway could be a key mechanism in the anticancer activity of **6**. Collectively, this information may lead to consideration of whether cysteine sulfenylation could be the mechanism by which the pharmacological actions of **6** are expressed. For example, in the structure of the possible target CCL2, there are two adjacent cysteines that could be subject to sulfenylation. However, there are no reports in which this aspect has been studied in depth.

Another recently reported antitumoral mechanism of **6** is inhibition of the enzyme histone deacetylase.^76,77^ In breast tumors, cancer cells are usually located away from blood vessels in a hypoxic environment. In order to adapt to these hypoxic conditions, the cancer cells increase levels of HIFs, which induce the expression of multiple genes involved in angiogenesis, cell proliferation, resistance to apoptosis, invasion, and metastasis. Therefore, drugs that can decrease HIF activity could reduce primary tumor growth, vascularization, invasion, and metastasis in breast cancer.^78,79^ DAT (**6**) seems to be a potential HIF-1α inhibitor, since it can attenuate the metastatic potential of breast cancer MDA-MB-231 cells in hypoxia-induced embryonic zebrafish, xenograft, and orthotopic tumors and can efficiently inhibit HIF-1α expression.^80^ On the other hand, another report showed that **6** inhibited the expression of the α-secretases ADAM10 and ADAM17 in estrogen-independent MDA-MB-231 and estrogen-dependent MCF-7 breast cancer cells. These studies also found that **6** reduced colony formation in a dose-dependent manner.^81^ This garlic-derived compound was also tested in an experimental model of breast cancer in combination with doxorubicin (**21**), a chemotherapeutic agent. The synergistic treatment effect markedly decreased tumor volume and weight, increased animals’ survival rate, and attenuated doxorubicin-induced tumor inflammation.^82^ Given the urgent need to develop new therapies for breast cancer due to the development of resistance to current treatments, the combination of chemotherapy drugs with **6** could be an effective approach.

*In vitro* analysis of **6** against human synovial sarcoma SW928 cells demonstrated that **6** induced apoptosis and G2/M cell cycle arrest and increased intracellular ROS through a possible induced dysfunction of the microtubule network.^83^

Another target protein is sulfiredoxin (Srx), an antioxidant enzyme, which is overexpressed in a variety of cancers. It seems to promote carcinogenesis and tumor progression.^84,85^ DAT (**6**) can inhibit Srx expression and ROS levels in gastric cancer BGC823 cells.^86^ Likewise, **6** inhibits the proliferation of human gastric carcinoma AGS cells by promoting apoptosis and accumulation of cells in the G2/M phase through ROS-dependent activation of the AMPK pathway.^87^ In another study performed in BGC-823 cells, **6** also induced cell cycle arrest at the G2/M phase, with significant overexpression of cyclin A2 and B1, and apoptosis through the attenuation of Nrf2/Akt and activation of p38/JNK. Furthermore, intraperitoneal administration of **6** at different doses (20, 30, and 40 mg/kg) to BGC-823 xenografted BALB/c nude mice for 32 days demonstrated a dose-dependent efficacy with 38, 50, and 57% tumor growth inhibition, respectively. The combination of **6** with cisplatin (**22**) enhanced antitumoral activity with fewer side effects.^88^ The induction of apoptosis and cell cycle arrest in the G2/M phase by **6** has also been reported in anaplastic thyroid carcinoma 8580C cells.^89^ Thus, **6** has been shown to induce apoptosis and cell cycle arrest in the G2/M phase *in vitro* and *in vivo* in numerous types of cancers.

### 2.2 Natural Alkenyl Benzenes

**Figure 6:**
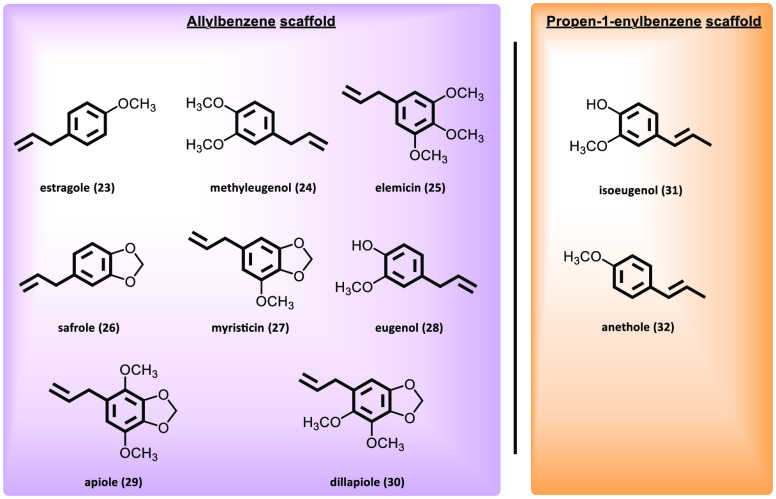
Chemical structures and names of natural alkenyl benzenes.

Alkenyl benzenes occur naturally in a wide variety of plants, including cinnamon (*Cinnamomum burmannii*), nutmeg (*Myristica fragrans*), and thyme (*Thymus vulgaris*). The most common alkenyl benzenes are estragole (**23**), methyleugenol (**24**), elemicin (**25**), safrole (**26**), myristicin (**27**), eugenol (**28**), apiole (**29**), dillapiole (**30**), isoeugenol (**31**), and anethole (**32**) (Figure 6). In contrast to garlic-derived compounds, which contain at least one thioallyl group in their structure, the alkenyl benzenes possess an allylbenzene scaffold. Isoeugenol (**31**) and anethole (**32**) contain the propen-1-enyl substituent in place of the allylic chain.

**Figure 7:**
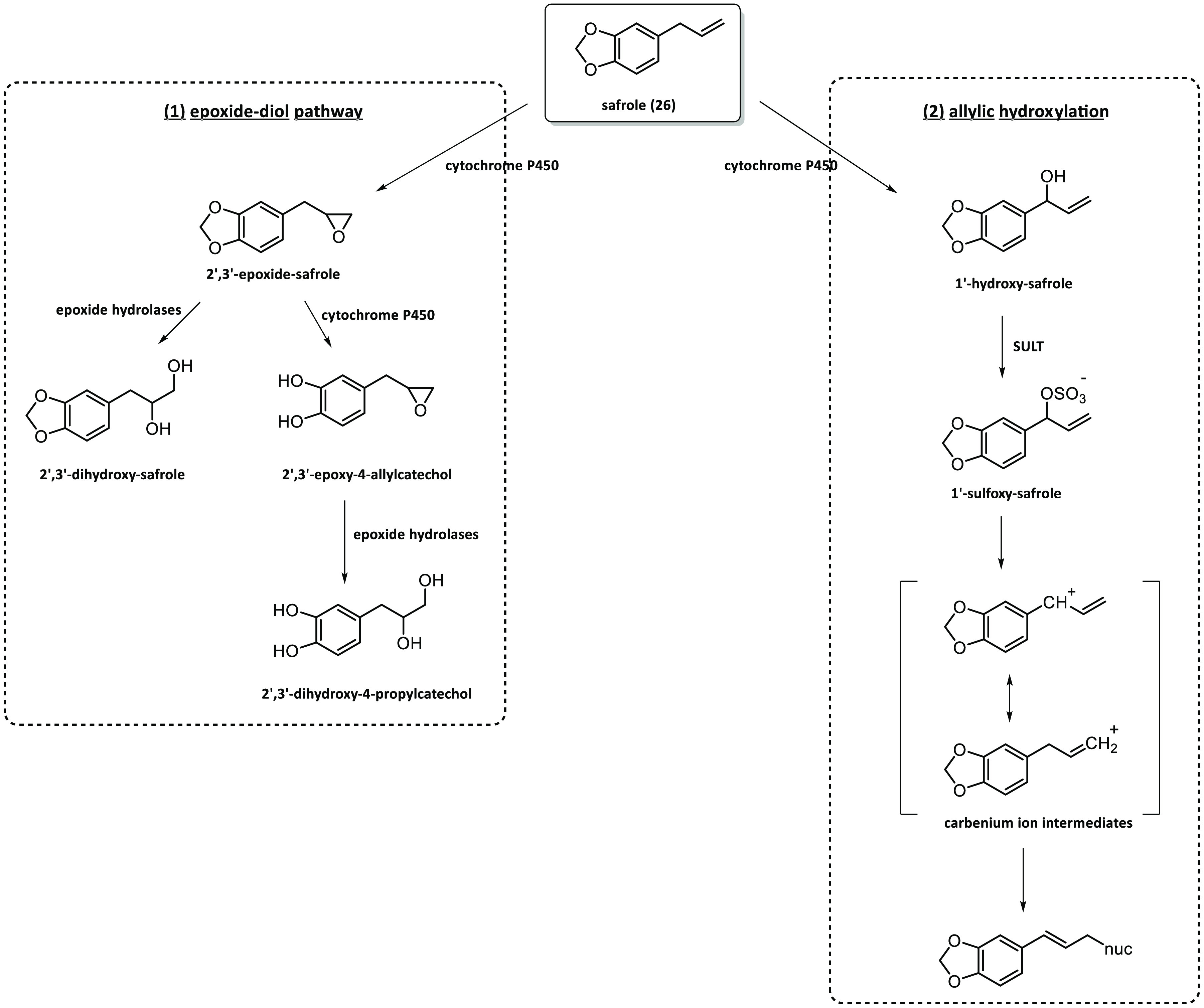
Metabolic pathways of alkenyl benzenes: (1) epoxide-diol pathway and (2) allylic hydroxylation. Modified from Götz et al.^97^

These molecules express different biological activities that include antitumor, analgesic, and antimicrobial effects.^90−92^ Estragole (**23**), methyleugenol (**24**), safrole (**26**), and anethole (**32**) have been shown to be hepatotoxic, genotoxic, and carcinogenic, unlike myristicin (**27**) and elemicin (**25**), which have demonstrated no carcinogenic potential *in vitro* and *in vivo*.^93−96^ However, the studies performed to date are not conclusive, and it cannot be stated that alkenyl benzenes are non-carcinogenic.^97^ Scientific evidence points to their metabolites as possibly responsible for their genotoxicity and carcinogenicity. The alkenyl benzenes share common features during the initial steps of hepatic activation that encompasses two main metabolic pathways: (1) epoxidation of the exocyclic double bond and (2) hydroxylation at the 1′-position, leading ultimately to the sulfoxymetabolites (Figure 7). These reactions of the side chains of alkenyl benzenes are catalyzed by several cytochrome P450 monooxygenases (CYP450). The epoxide group, after the action of epoxide hydrolases, gives rise to the 2′,3′-dihydrodiols. Moreover, those alkenyl benzenes that contain a methylenedioxy moiety can undergo demethylenation to yield a catechol. Metabolism of phenolic and catecholic compounds can proceed through rapid phase II conjugation, which could be a predominant pathway for these metabolites, or bioactivation to *ortho*-quinones. This point may explain why eugenol (**28**) appears to be less toxic, as it has a free phenolic group and exhibits high first-pass conjugation and rapid elimination.^97−99^

**Figure 8:**
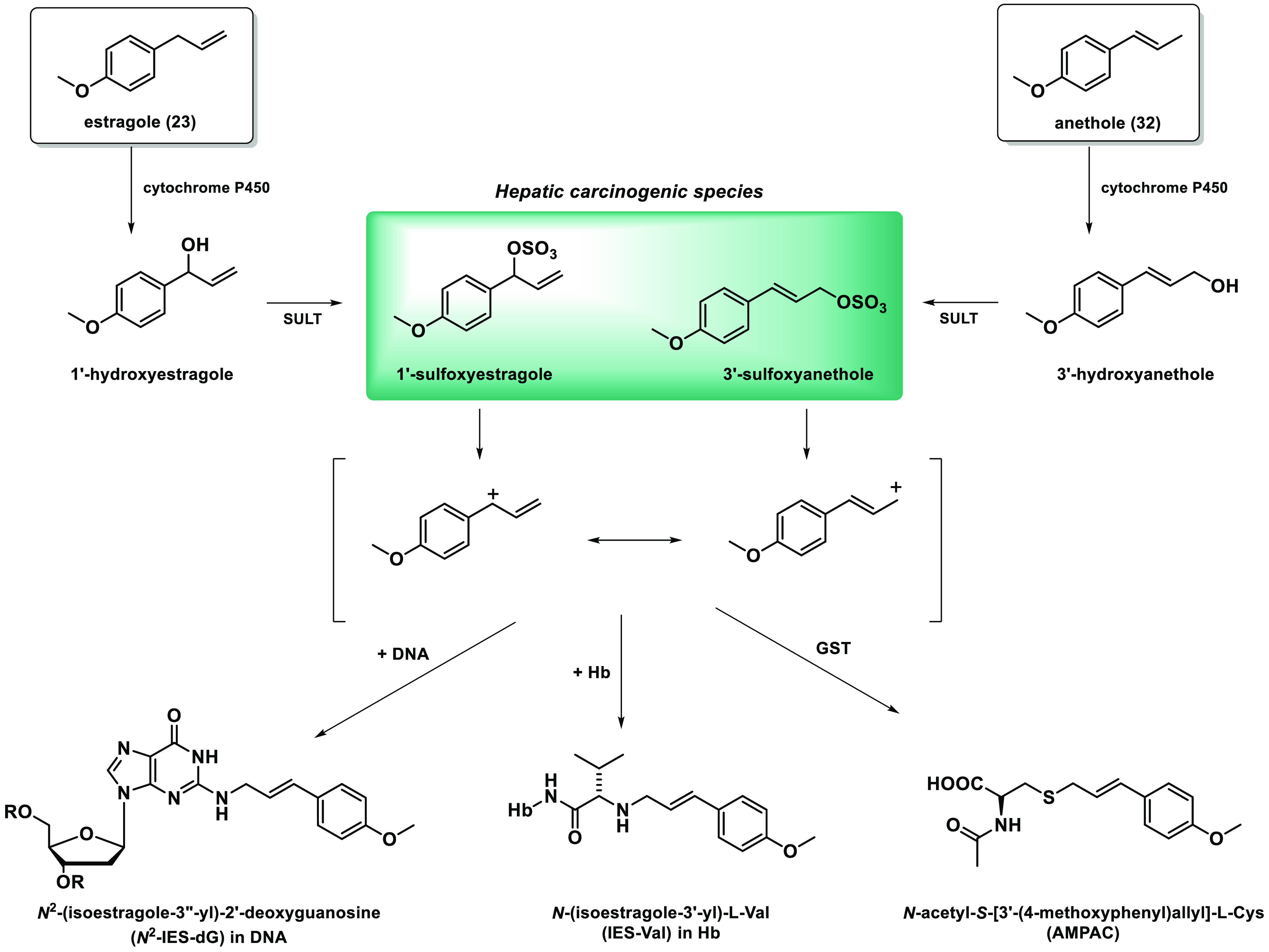
Bioactivation of estragole and anethole catalyzed by cytochrome P450 and sulfotransferases (SULTs) to the respective sulfuric acid esters. Modified from Bergau et al.^100^

The connectivity between the allyl chain and the benzene ring renders the CH_2_ as both an allylic and a benzylic carbon, resulting in a very stabilized carbocation when alkenyl benzenes undergo metabolic hydroxylation at the benzylic carbon. Both estragole (**23**) and anethole (**32**) present activated allylic positions where metabolic hydroxylation can occur. These positions are benzylic and allylic for the first one and just allylic for the second one. Subsequently, the hydroxylated species can be further metabolized by sulfotransferases to form the sulfate esters, which are carcinogens. However, they should have a very short lifetime, given that they present a very good leaving group and the stability of the carbenium ion is great but also highly reactive toward nucleophilic proteins. Thus, adducts with DNA, hemoglobin (Hb), or glutathione *S*-transferase (GST) may be formed (Figure 8).^100^

By comparing the structures of the alkenyl benzenes, the only difference between eugenol (**28**) and isoeugenol (**31**) is the position of the double bond. As we have just mentioned, the double bond in eugenol (**28**) is terminal and has a very reactive methylene group. In contrast, in isoeugenol (**31**) the double bond is located on the carbon attached to the aromatic ring, leaving a terminal methyl group, which is less reactive. Hence, eugenol (**28**) should be more reactive toward epoxide-diol and allylic hydroxylation pathways (Figure 7). The same applies to estragole (**23**) and anethole (**32**). Could this structural variation be the cause of a difference in activity? In the MCF-7 cell line after 48 h of treatment, **28** has shown an IC_50_ value higher than 1500 μM,^101^ whereas the IC_50_ value for **31** was 11.14 μM.^102^ However, there is no further information available to draw any conclusions. It is therefore an interesting question to address in the future.

As the genotoxic activity of alkenyl benzenes requires hepatic bioactivation, the use of nanotechnology emerges as a promising strategy to reduce this problem and to allow the use of these natural molecules for the treatment of several diseases. The small size (∼100 nm) of nanoparticles allows them to cross cellular membranes and avoid detection by the reticuloendothelial system in the liver, thus interfering with their metabolism. Moreover, there are several strategies that may be used to avoid hepatic metabolism. Among them are surface modification of nanoparticles with polyethylene glycol (PEG), which has long been a standard approach to reduce phagocytosis and improve tumor accumulation. However, it presents disadvantages, including the development of anti-PEG antibody response.^103^ On the other hand, the surface of the nanoparticles can be easily modified through the addition of targeting ligands such as peptides, proteins, or antibodies. These ligands enable selective uptake into tumor cells, increasing efficacy and avoiding hepatic metabolism. Table 2 summarizes the antitumoral activity of the allylbenzene derivatives.

**Table 2:** Modes of Action of Natural Alkenyl Benzenes and Allyl Isothiocyanate Compounds in Cancer

Type of cancer	Mode of action	refs
**Myristicin (27)**		
Hepatic (Huc-7 and HCCLM3)	Inhibition of cell proliferation	(111)
	Apoptosis	
	Suppression of cell migration and invasion by inhibition of EMT (↑ E-cadherin and ↓ N-cadherin)	
	Suppression of PI3K/Akt/mTOR signaling pathway	
Leukemia (K562)	Mitochondrial mediated apoptosis (release of cytochrome c and activation of caspase-3)	(115)
	PARP cleavage	
	DNA fragmentation	

***Myristica fragrans* Houtt. Extract**		
Epidermal (KB)	Apoptosis through decrease bcl-2 expression	(117)
	Inhibition of cell proliferation	

**Safrole (26)**		
Osteosarcoma (MG63)	[Ca^2+^]i increase	(119)
	Reduce cell viability	
Oral (HSC-3 *in vitro* and xenograft athymic nu/nu mouse model)	Caspase-dependent apoptosis	(120)
	Inhibition of tumor growth	
Leukemia (HL-60)	G0/G1 cell cycle arrest by inhibition of cyclin E	(122)
	Apoptosis through endoplasmic reticulum stress and mitochondrial-dependent pathway	
Leukemia (WEHI-3 xenograft BALB/c mice model)	Enhance humoral immune response, cellular immune response, and NK cell cytotoxicity	(123)
Liver (Hep3B)	Cytotoxicity	(92)

**Safrole Nanoemulsion**		
Liver (Hep3B)	Cytotoxicity	(92)

**Anethole (32)**		
Osteosarcoma (MG-63)	GI_50_ value of 6.25 μM	(129)
	Apoptosis through the mitochondrial mediated pathway	
	Cell cycle arrest at the G0/G1 phase	
	Up-regulates the expression of p53, caspase-9/-3 and down-regulates Bcl-xL expression	
Oral (Ca9–22)	Cell proliferation inhibition	(130)
	Apoptosis and autophagy	
	Decreases ROS production and increases glutathione activity	
	Inhibits cyclin D1 oncogene expression, increases cyclin-dependent kinase inhibitor p21WAF1, up-regulates p53 expression and inhibits EMT markers.	
	NF-kB, MAPkinasas, Wnt, caspase-3, -9 and PARP1 pathways involved	
Breast (MFC-7 and MDA-MB-231)	Apoptosis at 10^–3^ M	(131)
	Suppresses cell survival through an ER independent manner	
	Induction of caspase-9 and PARP1/2 cleavage	
	Elevation of c-FLIP and p53 expression	
Prostatic (PC-3)	Inhibition of cell proliferation, clonal growth, and migration	(132)
	Apoptosis by mitochondrial and lysosomal membrane permeabilization, caspase-3 and -9 activation, DNA damage, PARP cleavage, and increase of Bax/Bcl-2 ratio	
	ROS generation	
	G2/M cell cycle arrest	
	Reduction of cyclins proteins D1, CDK-4 and c-Myc and up-regulation p21 and p27 expression	
	Suppression of nuclear localization of NF-kB protein and down-regulation of transcription of NF-kB-dependent genes	

**Anethole + Doxorubicin**		
Breast (MDA-MB-2341)	Mitochondrial-mediated apoptosis by modulating Bax/Bcl-2 expression and activation of caspase-3	(133)
	Increase of intracellular ROS	
	Cell cycle arrest at the G2/M phase and S phase	

**Eugenol (23)**		
Breast (MDA-MB-2341 and SK-BR-3)	Autophagy and apoptosis via PI3K/AKT/FOXO3a pathway	(139)
	Caspase-mediated apoptosis	
Breast (CAF)	Suppresses the migratory and proliferative potential and their Paracrine pro-carcinogenic effect	(140)
	Modulates the methylation pattern and inhibits the expression of DNA Methyltransferase genes DNMT1 and DNMT3A	(144)
Gastric (AGS)	p53-, p21- and SMAD4-independent anti-metastatic activity through inhibition of TGF-β signaling	(145)

**Eugenol + Doxorubicin**		
Breast (MCF-7)	Apoptosis through up-regulated Bax/Bcl-2 ratio	(142)

**Allyl Isothiocyanate (36)**		
Colon (HT-29)	Apoptosis through ROS-based ER stress and mitochondria-dependent pathway	(151)
	Cell cycle arrest in the G2/M phase	
Breast (MCF-7)	Apoptosis through induction of DNA damage and alteration of DNA repair	(152)
Prostatic (RV1 and PC3)	Apoptosis	(153)
	Autophagy mediated by the up-regulation of beclin-1	
Oral (CAL27-cisplatin-resistant)	Inhibition of Akt/mTOR signaling	(154)
	Induction of mitochondria-dependent apoptosis through up-regulation of caspases-3 and -9	

Myristicin (**27**) is the major component of nutmeg (*Myristica fragrans*) and is found in smaller proportions in other species such as *Anethum graveolens* (dill) and *Petroselinum crispum* (parsley). Myristicin (**27**) is reported to have several pharmacological properties, including antioxidant,^104,105^ antimicrobial,^106,107^ antidiabetic,^108,109^ anticancer,^109−111^ anti-inflammatory,^107^ and antidepressant^112^ activities. However, when it is used in very high amounts (400 mg or more), **27** can express toxic effects, leading to liver degeneration and mental confusion, as it is toxic to the central nervous system. The toxic effects of **27** are thought to be related to its capacity to inhibit the enzyme monoamine oxidase (MAO), and it has been suggested that it is able to modulate GABA receptors, thereby generating anxiety.^113,114^

The role and related molecular mechanism of **27** in HCC Huc-7 and HCCLM3 cell lines were studied, and it was revealed that not only could **27** inhibit cell proliferation and induce apoptosis but it also suppressed cell migration and invasion. Myristicin (**27**) inhibited the EMT by increasing *E*-cadherin and decreasing *N*-cadherin expressions. Finally, suppression of the PI3K/Akt/mTOR signaling pathway was proposed as a mode of action.^111^ Myristicin (**27**) can also induce mitochondrial-mediated apoptosis in human leukemia K562 cells in a dose-dependent manner at concentrations ranging from 50 μM to 200 μM, with the release of cytochrome *c* and the activation of caspase-3. Additionally, PARP cleavage and DNA fragmentation (after 48 h at a concentration of 50 μM and above), with down-regulation of DNA damage response genes, have been reported.^115^ On the other hand, direct genotoxicity, repair, and apoptotic activities of **27** in mammalians AA8 and EM9 cells have also been studied. Myristicin (**27**) was shown to induce caspase-mediated apoptosis at a concentration of 750 μM after 24 h in both the cell lines, being more significant in EM9, and was not genotoxic in either cell line in the comet and in C-H2AX assays. The MTT assay carried out at different concentrations (from 50 to 2000 μM) after 24 h of treatment showed a reduction in cell viability.^116^ In addition to testing **27** alone, *Myristica fragrans* extract has been evaluated against the human oral epidermal carcinoma KB cell line and shown to inhibit cell proliferation with an IC_50_ value of 75 μg/mL. The extract was also able to decrease Bcl-2 expression, inducing early and late apoptosis at the concentration of 100 μg/mL.^117^

Safrole (**26**) is the major component of sassafras root extract. The main sources of safrole are *Ocotea odorifera*, *Piper auritum*, and *Sassafras albidum*. It is also the main component of brown camphor oil. Its structure includes a benzodioxole ring and a pendent allylic chain in position 2. It is a known carcinogen that can bind to DNA to form adducts at high concentrations after metabolic activation. The metabolic pathways for forming adducts of **26** are depicted in Figure 7.^118^ However, there are several reports on the anticancer activity of **26**, which markedly increases intracellular calcium concentrations (EC_50_ value of 450 μM) and decreases cell viability (dose of 650 μM) in human osteosarcoma cells.^119^ Moreover, the ability of **26** to induce caspase-dependent apoptosis and reduce cell viability in human oral squamous cell carcinoma has also been reported. *In vivo* results of a xenograft mouse model supported the *in vitro* results in the oral cancer HSC-3 cell line, as **26** could reduce the size of oral tumors at the dose of 15 mg/kg.^120^ However, xenograft models appear not to be the most adequate to represent oral cancer. On the other hand, other evidence showed the capacity of **26** to cause a 50% increase in cell proliferation in human oral cancer OC2 cells at the concentration of 10 μM.^121^ Thus, an in-depth study is needed to further establish the antitumoral capacity of **26** in oral carcinoma. Additionally, **26** has been evaluated against human leukemia HL-60 cells, and it showed anticancer activity by different mechanisms. It provoked G0/G1 phase arrest via inhibition of cyclin E and induced apoptosis by ER stress and a mitochondrial-dependent pathway.^122^ It has also been studied as an immunological adjuvant in leukemia. At low doses (less than 16 mg/kg) in leukemic BALB/c mice, **26** enhanced humoral immune and cellular immune responses, as well as increased NK cell cytotoxicity.^123^ As mentioned, safrole is a hepato-carcinogenic compound, which may limit its use as a therapeutic molecule. Therefore, to reduce its toxicity, a nanoemulsion formulation of safrole was developed which was evaluated against different biological targets. Its cytotoxicity at different concentrations was analyzed against Hep3B cancer cells using the MTT assay. The results showed a higher cell inhibition (87.25%) by safrole nanoemulgel when compared with the safrole oil (75.72%), with IC_50_ values of 0.31 mg/mL and 1.08 mg/mL, respectively.^92^

Anethole (**32**) is a phenylpropanoyl compound, and two isomers can be found in nature, *Z*-anethole and *E*-anethole. It contains the propen-1-enyl substituent in place of the allylic chain, and it is present in anise and fennel, providing a major component of their flavor and odor. The use of anethol suffered a pause due to concerns about its safety, since hepatic toxicity and possible carcinogenic activity in rats had been reported.^124,125^ However, the evidence is scanty, and the Flavor and Extract Manufacturers Association (FEMA) of the USA has classified it as “generally recognized as safe” (GRAS).

Several studies have reported its pharmacological activities, including anti-inflammatory,^126^ antimicrobial,^127,128^ and antitumoral effects.^129^ The effect of *E*-anethole was studied in the osteosarcoma MG-63 cell line, and the antiproliferative activity was evaluated by an MTT assay. It showed a GI_50_ value of 60.25 μM with apoptosis induction through the mitochondrial-mediated pathway. Additionally, it induced cell cycle arrest at the G0/G1 phase, up-regulated the expression of p53, caspase-3, and caspase-9, and down-regulated Bcl-xL expression.^129^ Moreover, the antitumoral activity of anethole was assessed against oral tumor Ca9-22 cells, and the cytotoxic effects were evaluated by MTT and LDH assays. It demonstrated a LD_50_ value of 8 μM, and cellular proliferation was 42.7% and 5.2% at anethole concentrations of 3 μM and 30 μM, respectively. It was reported that it could selectively and in a dose-dependent manner decrease cell proliferation and induce apoptosis, as well as induce autophagy, decrease ROS production, and increase glutathione activity. The cytotoxic effect was mediated through NF-kB, MAP kinases, Wnt, caspase-3 and -9, and PARP1 pathways. Additionally, treatment with anethole inhibited cyclin D1 oncogene expression, increased cyclin-dependent kinase inhibitor p21WAF1, up-regulated p53 expression, and inhibited the EMT markers.^130^

In breast cancer cells, **32** was able to suppress cell survival, induce apoptosis, and repress NF-kB transcriptional activity (MCF-7 and MDA-MB-231 cells).^131^ Likewise, on the prostate cancer PC-3 cell line, **32** inhibited proliferation, clonal growth, and migration, in addition to suppressing the growth of PC-3-derived CSCs (tumorspheres). In the study of the molecular mechanism, a pro-apoptotic activity was demonstrated along with ROS generation, mitochondrial and lysosomal membrane permeabilization, caspase-3 and -9 activation, DNA damage, PARP cleavage, and increase of Bax/Bcl-2 ratio. Moreover, additional mechanisms have been determined, including induction of G2/M phase arrest, reduction of cyclins proteins D1, CDK-4, and c-Myc, up-regulation of p21 and p27 expressions, and suppression of nuclear localization of NF-kB protein.^132^

As mentioned above, the combination of phytochemicals with FDA-approved chemotherapeutic agents has been widely studied. Anethole (**32**) has been evaluated in combination with doxorubicin against MDA-MB-231 TNBC cells. Combination treatment (50 μM of **32** and 0.5 μM of **21**) showed enhanced cytotoxicity, along with augmented inhibition of colony formation and migratory capacity. Cell viability was around 60% and 40% for single treatments with **32** (50 μM) and doxorubicin (0.5 μM), respectively, while the combination treatment showed a cell viability value of 30%. Loss of mitochondrial membrane potential, an increase in ROS production, and cell cycle arrest at both G2/M and S phases were also reported. Treatment with **32** alone demonstrated its potential to promote apoptosis, but when combined with doxorubicin, it promoted enhanced mitochondrial-mediated apoptosis by modulating Bax/Bcl-2 expression and activation of caspase-3.^133^ These results give rise to studying this combination of drugs *in vivo*, as it could be the starting point to be able to take this synergistic treatment to the clinic, with a decrease in the dose of doxorubicin and therefore its toxicity.

**Figure 9:**
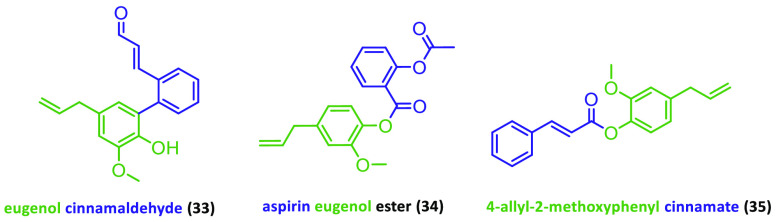
Eugenol-based molecules with the highest binding to breast cancer receptors.

Eugenol (**28**), the most studied natural alkenyl benzene, is present in large quantities in the essence of cloves (*Syzgium aromaticum*). Its alkenyl benzene structure contains a methoxy group in the *meta*-position and a hydroxyl group in the *para*-position. It is commonly used in dentistry as a temporary filling material when mixed with zinc oxide,^134,135^ as well as a pulp sedative, temporary cementing agent, and dental protector, among others. Beneficial roles of **28** in modulating oral inflammation,^136^ pain reduction,^137^ and oral wound healing^138^ have been reported. Additionally, the effect of **28** on cancer has been widely studied, with breast cancer being one of the most widely assessed. For example, **28** was evaluated against triple-negative (MDA-MB-231) and HER2-positive (SK-BR-3) breast cancer cell lines, demonstrating both apoptosis and autophagy. Pro-apoptotic proteins, including caspase-3 and -9, p21, p27, AKT, and FOXO3a, were up-regulated in treated cells, as well as autophagy proteins. Inhibition of cell proliferation by more than 90% in both cell lines was indicated at 40 μM and 60 μM concentrations.^139,140^ In addition, eugenol derivatives (Figure 9) demonstrated high binding affinities to breast cancer receptors such as ERα, progesterone receptor, and cyclin-dependent kinase 2.^141^ Therefore, future *in vitro* and *in vivo* studies are needed to confirm the activity of these eugenol molecules in breast cancer.

Similarly, the combined treatment of doxorubicin and **28** has demonstrated a synergistic effect on inhibiting the proliferation of breast cancer MCF-7 cells. **21** alone showed an IC_50_ value of 0.5 μM, whereas its IC_50_ value in combination treatment with 1 mM of **28** was 0.088 μM, evidence of an *in vitro* synergistic effect of **28** with doxorubicin against breast cancer MCF-7 cells. In addition to this cytotoxic effect, *in vitro* treatment with the drug combination was able to produce epigenetic histone acetylation and immunomodulation of different apoptotic approaches.^142^ Thus, it seems that synergistic treatment might allow to an optimal concentration to be reached *in vivo*.

The search for new molecules targeting cancer-associated fibroblasts (CAFs) is a novel approach to treating cancer, as their roles in tumor onset, progression, and metastasis, as well as in cancer resistance and recurrence, have been demonstrated. Additionally, CAFs are the most active and abundant components of breast cancer stroma, and they have a paracrine pro-carcinogenic effect.^143^ In this context, **28** arises as a promising agent, as it can suppress the migratory and proliferative potential of breast CAF cells and their paracrine pro-carcinogenic effect by down-regulating E2F1 expression. Eugenol (**28**) modulates the methylation pattern and inhibits the expression of DNA methyltransferase genes DNMT1 and DNMT3A at a concentration of 1 μM in breast CAF cells.^144^ This concentration appears to be a good starting point to make the leap to *in vivo* experiments. Thus, the *in vitro* results position **28** as a promising molecule capable of modulating the epigenetic deregulation of breast CAF and providing a new therapy for these complex and heterogeneous tumors. Furthermore, **28** has demonstrated p53-, p21-, and SMAD4-independent anti-metastatic activity in gastric cancer AGS cells through the inhibition of TGF-β signaling at the dose of 66 μg/mL.^145^

Eugenol (**28**) can exert its chemotherapeutic effect by the degradation of β-catenin via *N-*terminal Ser32 phosphorylation *in vivo* and *in vitro* in lung and breast CSCs populations.^146,147^ Considering that the Wnt/β-catenin signaling plays a pivotal role in the self-renewal and maintenance of CSCs, which are the most resistant and virulent subpopulation of cancer cells, eugenol (**28**) should be considered as a potential candidate to treat lung and breast cancer, although further studies are needed.^71,72,148^

The results obtained in the evaluation of alkenyl benzenes as potential anticancer agents have mainly shown their ability to reduce cell viability and induce apoptosis in a wide variety of tumor cell lines, including some *in vivo* studies.

### 2.3 Allyl Isothiocyanate and Cancer

**Figure 10:**
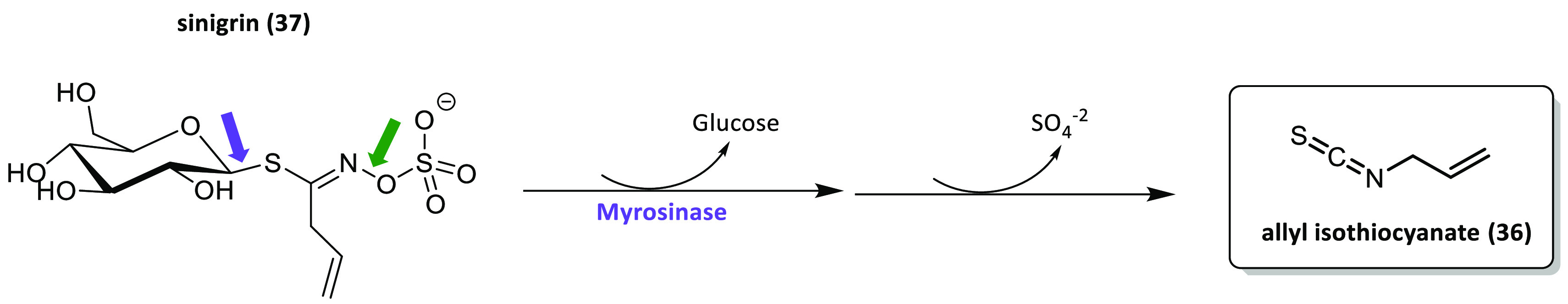
Enzymatic processing of sinigrin by myrosinase to release allyl isothiocyanate.

Allyl isothiocyanate (**36**) is a phytochemical that has been extensively studied as an anticancer agent.^149,150^ It is obtained when the seeds of black mustard (*Brassica nigra*) or brown Indian mustard (*Brassica juncea*) are broken, releasing the enzyme myrosinase, which acts on sinigrin, a glucosinolate, to form **36**, as depicted in Figure 10.

Allyl isothiocyanate (**36**) has a cytotoxic activity in cancer cell lines mainly mediated by the induction of apoptosis. In colon cancer HT-29 cells, **36** induced apoptosis through ROS-based ER stress and a mitochondria-dependent pathway, in addition to inducing cell cycle arrest in the G2/M phase.^151^ In breast cancer MCF-7 cells, **36** demonstrated its apoptotic effect through induction of DNA damage and alteration of DNA repair.^152^ Additionally, in prostatic cancer RV1 and PC3 cells, **36** not only promoted apoptosis but also induced autophagy mediated by the up-regulation of beclin-1.^153^ Furthermore, this allylic compound showed potent cytotoxic activity against CAL27-cisplatin-resistant human oral cancer cells, including the inhibition of Akt/mTOR signaling and the induction of mitochondria-dependent apoptosis through up-regulation of caspases-3 and -9.^154^ Based on the above observations, **36** could be a good candidate for cancer treatment, as it has demonstrated *in vitro* cytotoxic activity by inducing apoptosis through different pathways in several cancer cell lines.

**Figure 11:**
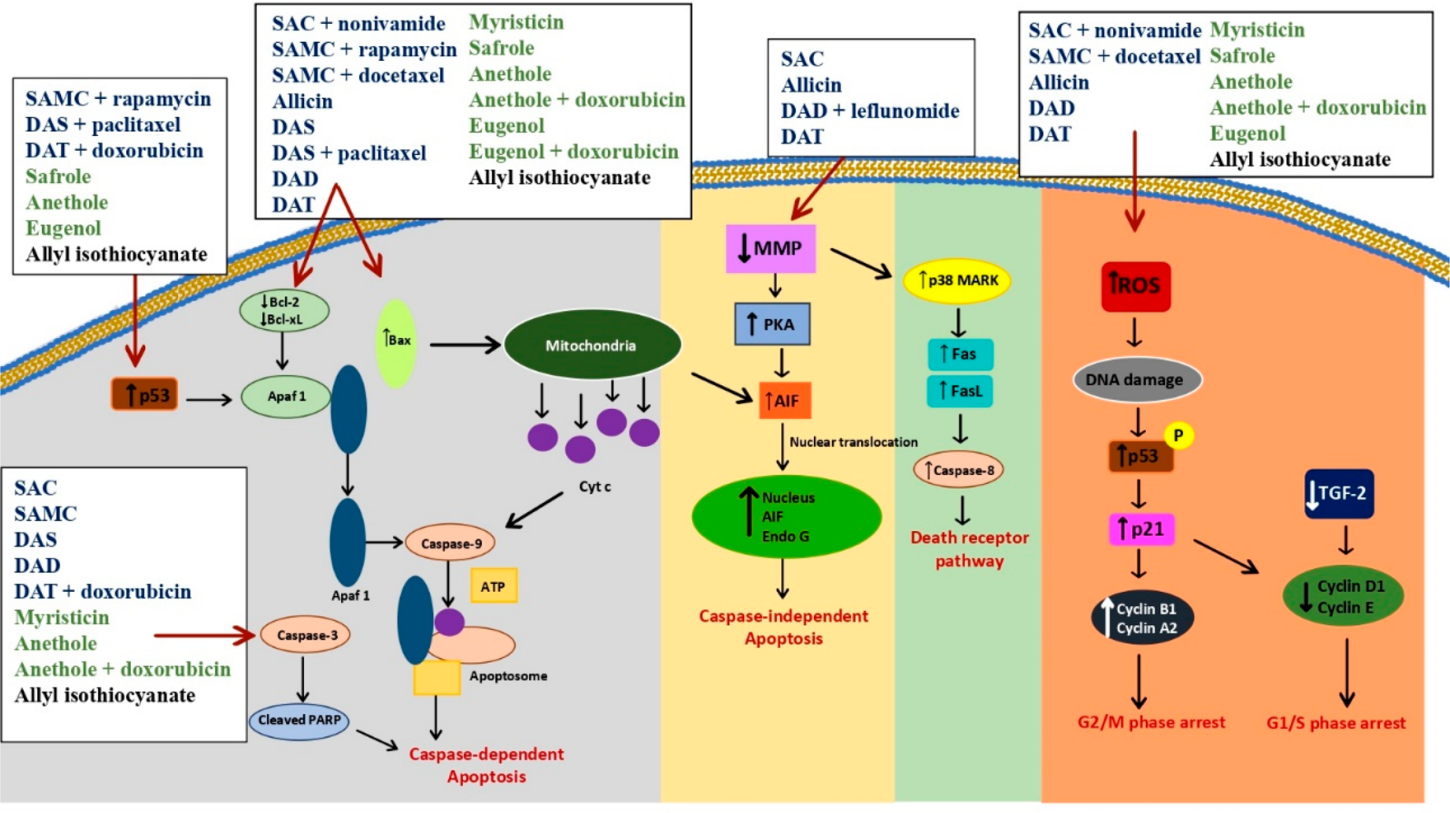
Natural allyl derivatives and their main mechanisms of action in cancer.

Figure 11 gathers the different mechanisms of action of natural allyl compounds in cancer.

## 3 Synthetic Allyl Compounds and Their Importance in Cancer Therapy

One of the most widely accepted approaches in drug development is the structural modification or introduction of bioactive molecules from NSs in novel therapeutic molecules to improve effectiveness and decrease toxicity.^155^ Probably one of the largest therapeutic areas in which the design of this hybrid molecule has been successfully applied is the field of anticancer agents. A multitude of new hybrid molecules have been developed combining FDA-approved drugs with molecules isolated from plants, such as vitamin E–paclitaxel,^156^ resveratrol–aspirin,^157^ and arimetamycin–doxorubicin.^158^ In other cases, the incorporation of active natural fragments, such as allyl residues in the synthesized compounds, has been used, and this will be discussed in this part of the Perspective. Table 3 summarizes the antitumoral activity of the most important synthetic allyl derivatives.

**Table 3:** Modes of Action of Synthetic Allylic Compounds in Cancer

Type of cancer	Mode of action	refs
**PAC-1 (38)**		
Leukemia (HL-60), lymphoma (U-937), melanoma (UACC-62, CRL-1782, B16-F10 and SK-MEL-5) neuroblastoma (SK-N-SH), breast (BT-20 and Hs 578t), lung (NCI-H226), adrenal (PC-12), and renal (ACHN)	Activating procaspase-3 to caspase-3	(159)
Primary cells of colon tumor	Apoptosis	
NCI-H226 and ACHN xenograft athymic BALB/c nude mice models		

**PAC-1 Derivative (39)**		
Colon (SW260), prostate (PC-3), and lung (NCI-H23)	Activating procaspase-3 to caspase-3	(160)
	Apoptosis	

**Curcumin Analogs (41 and 42)**		
Gastric (BGC823 and SGC-7901 *in vitro* and SGC-7901 xenograft mice model)	Cytotoxicity	(162,161)
	Apoptosis through Akt-FoxO3a	
	Cell cycle arrest at G2/M phase	
	Increases ROS	
	Activation of endoplasmic reticulum stress	
	Inhibition of STAT3 phosphorylation	

**17-AAG (49)**		
Breast, multiple myeloma, metastatic melanoma, renal, prostate and thyroid (Phase I, II/III clinical trials)	Binds to Hsp90 and destabilizes client proteins	(164)
	Decreases in HER2 and Pra-1, instability in p53, and interruptions in MAPK signaling	

**SMER28 (51)**		
Hepatic (hepG2) and normal NCTC cells and mice BALB/c mouse	Cytoprotection of normal tissues toward the sequelae of radiotherapy and chemotherapy	(180)
	Positive regulator of autophagy that is activated through an mTOR-independent mechanism	

**Compound 53**		
Prostate (DU-145 and PC-3)	Apoptosis and enhanced ROS formation	(185)
	DNA damage	

**Compound 58**		
Hepatocellular (HepG2)	Cytotoxicity activity	(189)
	Induce apoptosis	
	Cell cycle arrest at G1 phase	
	Inhibition of colony formation	

***N*-Benzoyl-3-allylthiourea (60)**		
Breast cancer with HER2+ (MCF7/HER2)	Cytotoxicity activity, enhances of HER2 expression, and inactivates NF-kB transcription factors	(190)

**Figure 12:**
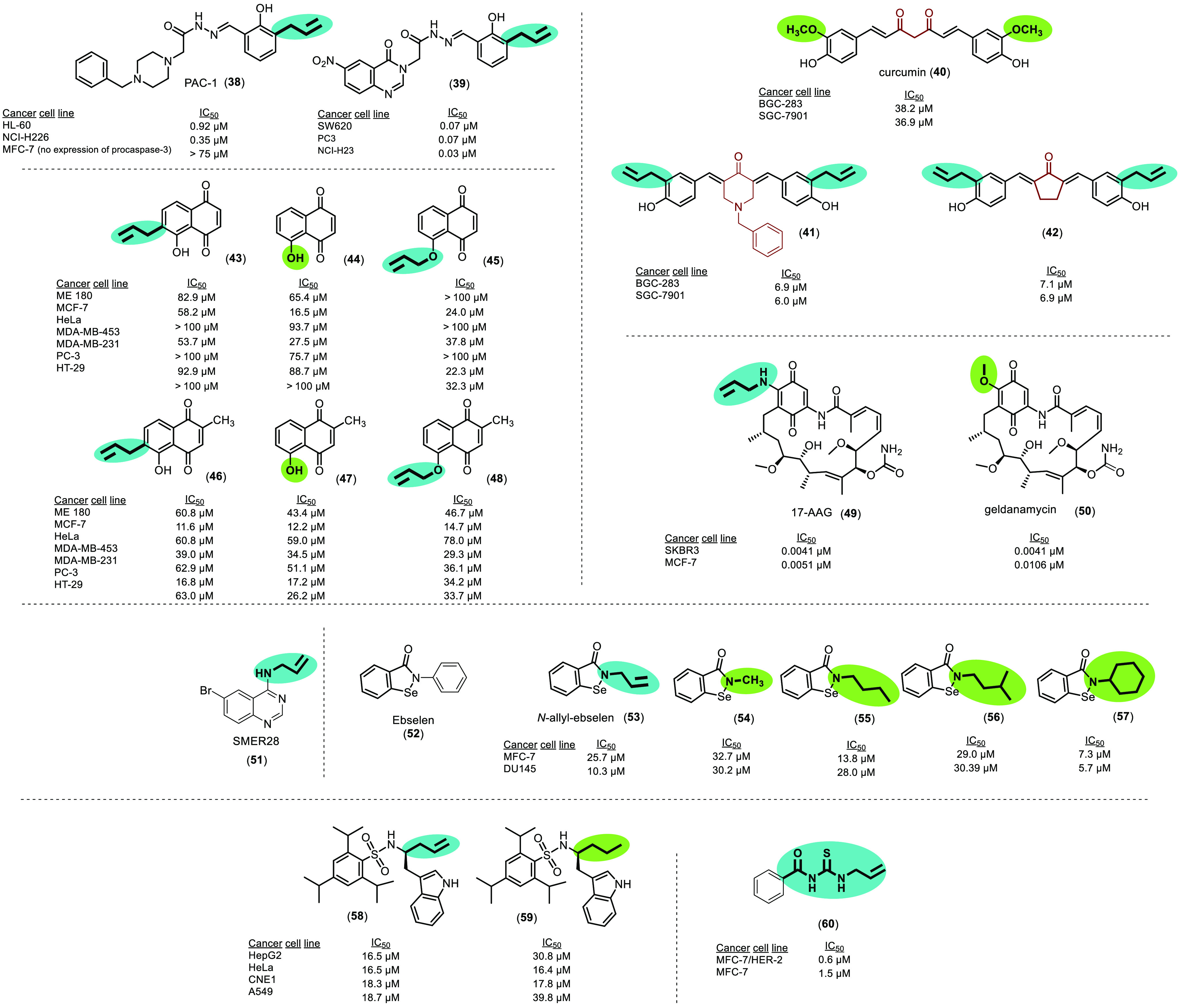
Chemical structures of synthetic allylic derivatives and their IC_50_ values against different cancer cell lines.

Considering the anticancer activity of natural alkenyl benzenes, several allylbenzene derivatives have been synthesized and evaluated. The procaspase-activating compound 1 (PAC-1, **38**), which is the first procaspase-activating compound that selectively induces apoptosis in cancerous cells, is an alkenyl benzene (Figure 12). It works by activating procaspase-3 to caspase-3 by chelating zinc, as procaspase-3 is known to be inhibited by low levels of this metal. PAC-1 (**38**) exhibits IC_50_ values between 0.003 μM and 1.41 μM in various cancer cells, while the IC_50_ values in non-cancerous cells were 7 times higher. *In vivo* studies performed supported *in vitro* observations by showing **38** to cause tumor regression.^159^ Given these promising results with **38**, a total of 13 derivatives were synthesized combining PAC-1 and 4-oxoquinazoline-based acetohydrazides. All of them showed cytotoxic activity, but **39** (Figure 12) stood out as the most active, being 213-fold more potent than 5-fluorouracil and 87-fold more potent than **38**. Additionally, in the caspase activation assay, **39** was able to activate it by 291% compared to **38**. Molecular docking studies suggested that **38** could be a potent zinc chelating agent, with the 2-hydroxy group and the acyl hydrazine moiety playing key roles for the formation of zinc chelates, which also suggests that the allyl chain is not specifically relevant for its activity.^160^

The allylated curcumin analogs **41** and **42** depicted in Figure 12 contain two allyl benzene groups in their structures. They showed potent *in vitro* anticancer activity, with IC_50_ values ranging from 6.0 μM to 7.0 μM against gastric BGC-283 and SGC-7901 cancer cell lines. In contrast, curcumin (**40**) exhibited lower cytotoxicity, with IC_50_ values of 38.2 μM and 36.9 μM, respectively, against the same cancer cell lines, 5-fold higher than those of the allylated analog.^161,162^ Significant cytotoxicity and induction of G2/M cell cycle arrest and apoptosis were demonstrated in gastric cancer cell lines. Additionally, in the *in vivo* assay, the allylated compounds reduced the growth of gastric cancer xenografts in tumor-bearing mice.

However, several series of compounds have also been synthesized in which allylic benzyl derivatives did not show significant *in vitro* anticancer activities. Among the results of these allylic compounds and their alkyl homologs, possible SARs are observed. Between **44**/**47** and **43**/**46**, the only difference is the allylic chain in the α-position relative to the hydroxyl group (Figure 12). *In vitro* cytotoxic activity was higher for **44**/**47**, which did not possess the allylic chain. The only allylic compound that showed cytotoxic activity was **46**, which was active in MCF-7 and PC-3 cell lines, although its IC_50_ values were comparable to those of **47** (its alkyl analog). Thus, the allylic chain at the α-position relative to the hydroxyl group did not appear to contribute to the anticancer activity. In addition, two new molecules, **45** and **48** (Figure 12), were synthesized by replacing the hydrogen of the hydroxyl group with an allylic chain. However, this modification did not enhance cytotoxic activity either.^163^

Another series of compounds with an *N*-allyl group in their structure, represented by 17-allylamino-17-demethoxygeldanamycin (17-AAG or tanespimycin; **49** depicted in Figure 12) and its derivatives, have been designed and synthesized. 17-AAG (**49**) is an analog of geldanamycin (**50**), an antitumor antibiotic that inhibits Hsp90, a chaperone whose up-regulation is associated with tumor progression, invasion, metastasis, and drug resistance. Despite the pharmacological activities of **50**, including its potent anticancer properties, it cannot be evaluated in clinical trials because of its hepatotoxicity, poor water solubility, and limited oral bioavailability. Therefore, analogs of **50** such as **49** have been developed. The only difference in the structures of **49** and **50** is the 2-substituent of the benzoquinone, which contains an *N*-allyl group instead of a methoxy group. This small change in the structure allows **49** to retain a potent anticancer activity with lower toxicity and better metabolic stability.^164^ In addition, **49** has been evaluated in Phase I and Phase II/III clinical trials for the treatment of multiple myeloma, metastatic melanoma, and renal and breast cancers.^165−169^ As with many other compounds, **49** has been given in combination with other drugs, including paclitaxel,^170^ rapamycin,^171^ trastuzumab,^168,172^ and irinotecan,^173^ and has demonstrated potent anticancer activity.^172,174−176^ Despite all the interesting results shown by **49**, its solubility in water, like that of **50**, remains low, making it difficult to formulate. Therefore, nanoformulations have been developed to solve this problem. 17-AAG (**49**) was loaded in β-cyclodextrins and evaluated against breast cancer T47D cells. The results showed that the β-cyclodextrin–**49** complex enhanced cytotoxicity and drug delivery, showing an IC_50_ value of 24 μg/mL compared to that of free **49**, which was 35 μg/mL.^177^ However, new formulations should be studied to achieve better outcomes. For the treatment of colon cancer, **49** was encapsulated in hybrid DOTA-PLGA nanoparticles decorated with hyaluronic acid (HA).^178^ HA was used because it has many favorable properties, including recognition of its receptor CD44, which is overexpressed in tumor-cell membranes.^179^*In vitro* and *in vivo* studies were performed and showed higher therapeutic efficacy of the **49**-loaded DOTA-PLGA nanoparticles than free **49**.

Another small molecule containing an *N*-allyl substituent is SMER28 (**51**, Figure 12), a positive regulator of autophagy that is activated through an mTOR-independent mechanism. It has been evaluated as a selective cytoprotector of normal tissues toward the sequelae of radiotherapy and chemotherapy. *In vitro* results confirmed that SMER28 enhanced autophagy and improved survival of normal hepatocytes, while no effect on hepatoma carcinoma cells was observed. Likewise, *in vivo* subcutaneous administration of this compound protected the liver and bone marrow of mice against radiation damage and facilitated their survival.^180^

Among the novel approaches for the design of new active compounds, the strategy of introducing selenium into their structure stands out. Se is a trace element characterized by its high antioxidant capacity. Ebselen (**52**), the most studied Se-containing compound, is a derivative of benzoselenazole and has shown a wide range of valuable biological functions, including anti-inflammatory, antioxidant, and cytoprotective.^181−183^ Thus, a series of ebselen analogs were developed as anticancer agents. All of the compounds differed in the amine substituent, with **53** substituted with an allyl chain. These compounds were tested against breast MFC-7 and prostate DU-145 cancer cell lines, with the result that there were no significant differences found between the allylated analog and the others. The compound with the highest cytotoxic activity was **57**, a cyclohexane derivative, while the allylated analog **53** was the second most active, highlighting an IC_50_ value of 10.3 μM against a prostate cancer cell line.^184^ In addition, **53** caused genotoxic stress in PC-3 cells, as significant DNA damage was observed. However, this was not found in PC-3 cells treated with the isopentyl derivative **56**. Finally, it was determined that **53** induced cell death through apoptosis and enhanced ROS formation.^185^ Thus, these studies show that the introduction of the allylic chain does not compromise cytotoxic activity but can actually enhance it and induce apoptosis. However, the fact that no significant difference in cytotoxic activity was found, with all derivatives showing IC_50_ values in the low–medium micromolar range, may be due to the Se-containing ring. This ring may interact with biologically important proteins. Thus, the anticancer activity of these analogs might be related to the presence of the Se pharmacophore and not to the different substituents used.^186−188^

A series of (*S*)-tryptamine derivatives containing an allyl (**58**) or propyl chain (**59**) (Figure 12) have been evaluated as anticancer agents. The allyl derivative **58** showed a more potent cytotoxic activity than **59** against the HepG2 cell line, the two compounds showing IC_50_ values of 16.5 μM and 30.8 μM, respectively, and against A549 cancer cell lines, with IC_50_ values of 18.7 μM and 39.8 μM, respectively. Additionally, **59** arrested the cell cycle at the G1 phase, induced apoptosis, and inhibited colony formation in HCC HepG2 cell line.^189^

In another study, the cytotoxicity of *N*-benzoyl-3-allylthiourea (**60**) was evaluated against HER2-overexpressed breast cancer MCF-7 cells. It exhibited a lower IC_50_ value (0.64 mM) in these overexpressed cells compared to regular breast cancer MCF-7 cells (1.46 mM). Although a decrease in IC_50_ value was observed, both values were in the millimolar range. Therefore, we do not believe that this allylthiourea derivative is a realistic inhibitor. On the other hand, molecular biology studies were carried out which revealed that **60** increased HER-2 expression and inactivated NF-kB transcription factors, resulting in inhibition of protein expression, which plays an important role in cell proliferation.^190^ However, further structural modifications should be studied to try to achieve a more potent inhibitor.

The literature collected in this section shows that compounds incorporating one or more allylic chains in their structure possess potent anticancer activity *in vitro* and *in vivo* in most cancers. For example, **38** has been shown to be a potent selective cytotoxic agent for cancer cell lines expressing procaspase-3. Its derivative, compound **39**, exhibited IC_50_ values in the nanomolar range and no activity against the non-tumor MRC-5 cell line (normal lung tissue fibroblasts). The incorporation of allylic chains in curcumin derivatives markedly improved the cytotoxic activity from IC_50_ values in the medium micromolar range to the low micromolar range. In addition, **41** could reduce both tumor volume and weight in a BGC-823 xenograft tumor mouse model, without histological alterations in the heart, liver, and kidney tissues of mice being noted. Incorporation of the allylic chain also enhanced the activity of the (*S*)-tryptamine derivative **58** in HepG2 and A549 cancer cell lines. However, in naphthoquinones **43**–**48** and the ebselen derivatives **53**–**57**, no significant differences were observed between the allylated and non-allylated derivatives. Even so, the allylated compound **46** showed activity against MCF-7 and PC-3 cell lines, and the allylic derivative of ebselen exhibited activity against prostate cancer DU145 cell line. Finally, it is worth mentioning the example of geldanamycin and **49**, in which the introduction of the allylic chain has managed to maintain the potent anticancer activity while improving solubility and decreasing hepatic toxicity. Furthermore, it is known that the introduction of allylic groups increases the lipid solubility of polar compounds, which is a very necessary character for the activity, since it improves their ability to cross cell membranes.

**Figure 13:**
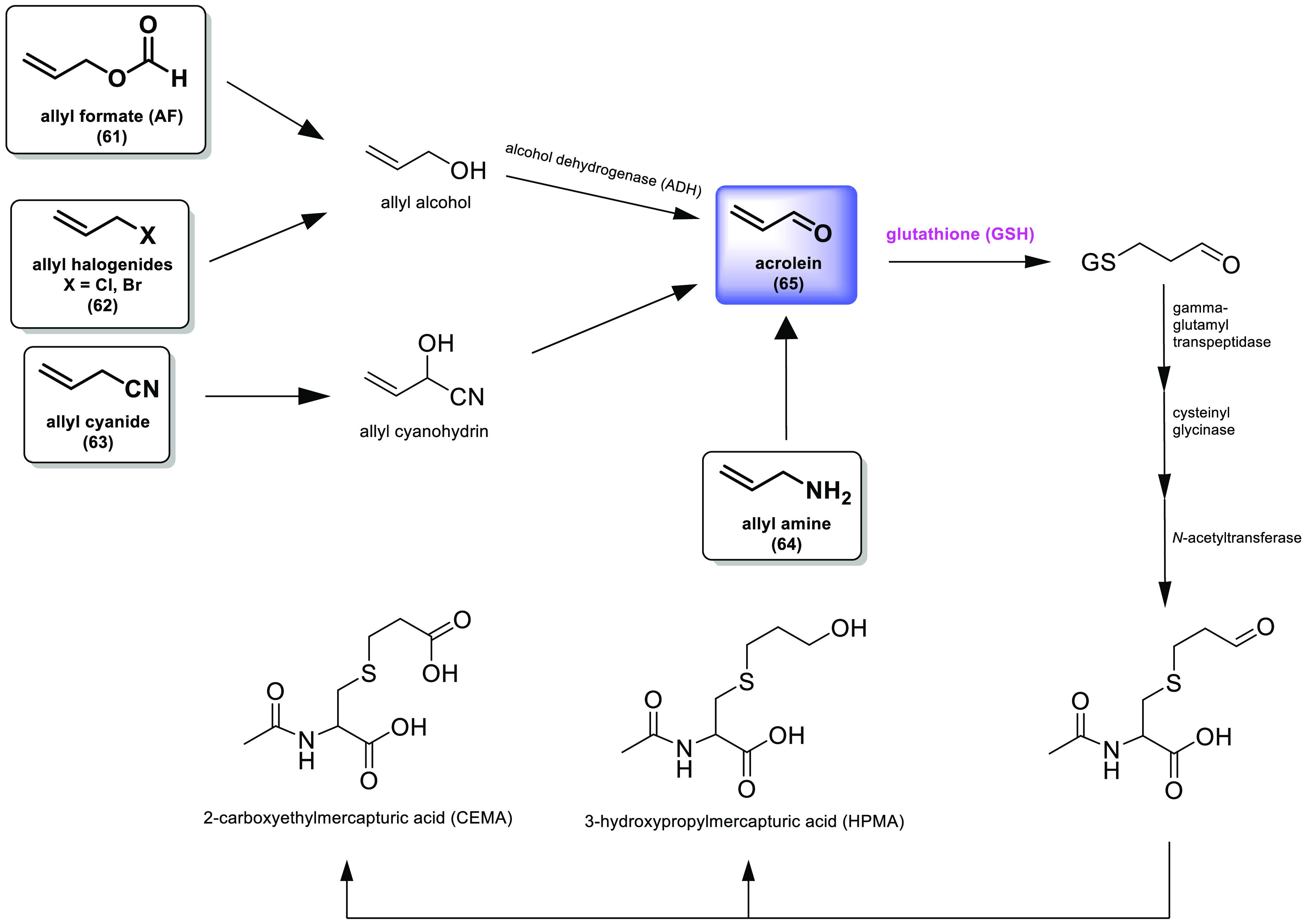
Detoxification pathway via glutathione conjugation with acrolein for certain allyl compounds. Modified from Athersuch et al.^192^

During phase I of metabolism, certain allyl derivatives may form some of the following metabolites, including allyl formate (**61**), allyl halogenides (**62**), allyl cyanides (**63**), and allyl amines (**64**). The intermediate metabolite of these compounds might be acrolein (**65**) (Figure 13). Acrolein (**65**) is classified by IARC as a group 2A carcinogen (probable human carcinogen). Currently, tobacco smoke is the main source of acrolein exposure. Acrolein (**65**) is conjugated with glutathione and excreted in the urine as metabolites of mercapturic acid. Like all molecules with electrophilic double bonds, acrolein can react with biological nucleophiles and produce undesirable results.^191^ Therefore, the potential toxicity of compounds with allylic regions will depend on many factors, the most important of which is the complete structure of the molecule, which will determine the detoxification route.

**Figure 14:**
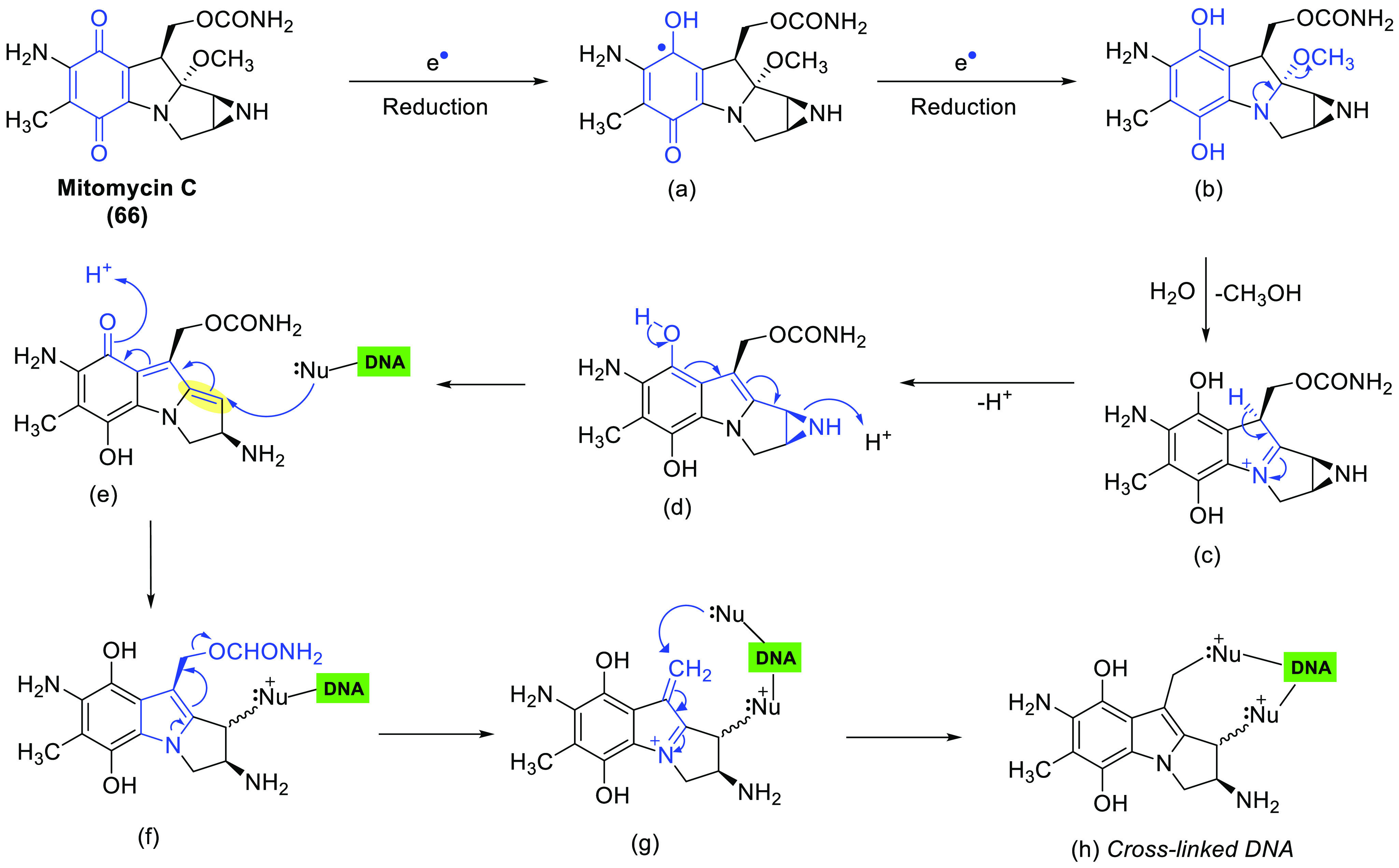
Bioreductive alkylation of DNA by mitomycin C. Modified from Avendaño et al.^193^

Mitomycin C (**66**), a DNA alkylating agent, is an example of a prodrug. It needs to be activated through *in situ* bioreductive activation to form an allylic derivative and exert its alkylating action. Intermediate e in Figure 14 contains an electrophilic position (the double bond) that reacts with nucleophilic groups on DNA via a Michael-type reaction to give the unstable intermediate f. This type of alkylating agent exhibits absolute specificity for N-7 of guanine and N-3 of adenine. However, despite the success of mitomycin C, its high toxicity remains a limitation for its clinical use.^193−195^

**Figure 15:**
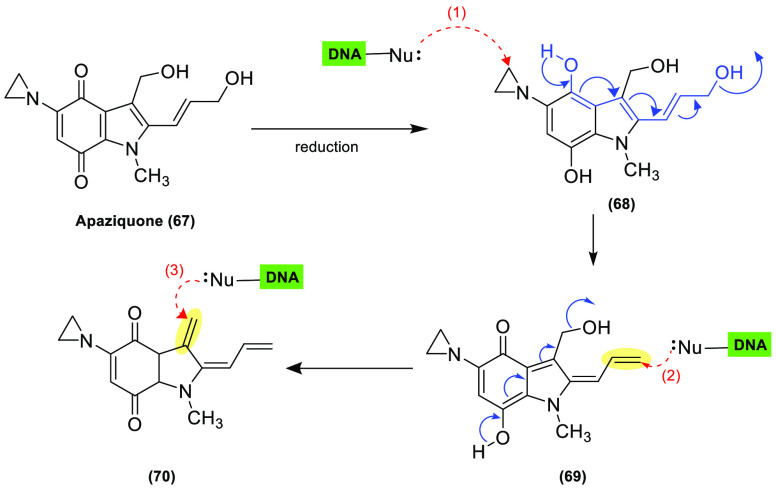
Bioreductive activation of apaziquone. Modified from Avendaño et al.^193^

In an attempt to reduce toxicity, new derivatives have been developed, including a library of compounds named EO. These compounds can form adducts with DNA through three different sites, by monoalkylation of the aziridine ring (position 1 in Figure 15) and cross-linking (positions 2 and 3 in Figure 13). Notable among these compounds is apaziquone or EO9 (**67**, Figure 15).

Similar to **66**, both carbonyl groups of the quinone ring can be reduced to form a hydroquinone intermediate. Then, the loss of a methanol molecule will yield an intermediate with a nitrogen atom positively charged. The subsequent electronic rearrangement activates the aziridine ring for nucleophilic attack by DNA.^193^ In addition, two elimination reactions take place to form the intermediates **69** and **70**, both with a highly electrophilic terminal alkyl double bond, which allows the alkylation of DNA. Apaziquone (**67**) has been highlighted, as it showed good activity against hypoxic cells and a lack of toxicity to bone marrow in preclinical models. The formation of its active metabolites takes place in hypoxic cells, mediated by intracellular reductases which are highly expressed in hypoxic tumor cells, enhancing its efficacy and decreasing its toxicity. It was therefore taken to clinical trials where it reached Phase II for breast, colon, pancreatic, gastric, and non-small-cell lung cancers. However, the very short half-life of the drug together with its poor tissue penetration prevented its systemic administration. These shortcomings became advantageous for local administration. Therefore, it was studied for early-stage superficial bladder cancer, where it demonstrated good activity and a lack of major organ toxicity. However, the Phase III clinical trial did not reach the expected objective, and development was discontinued.^196−198^

The case of **67** is an example of how the design of prodrugs could be beneficial to avoid the toxicity that may be associated with an allyl group. The use of an advantageous feature of tumor cells, such as the hypoxemic environment, for the design of prodrugs that require bioreduction in these hypoxic cells would be a breakthrough for the design of new anticancer drugs.

**Figure 16:**
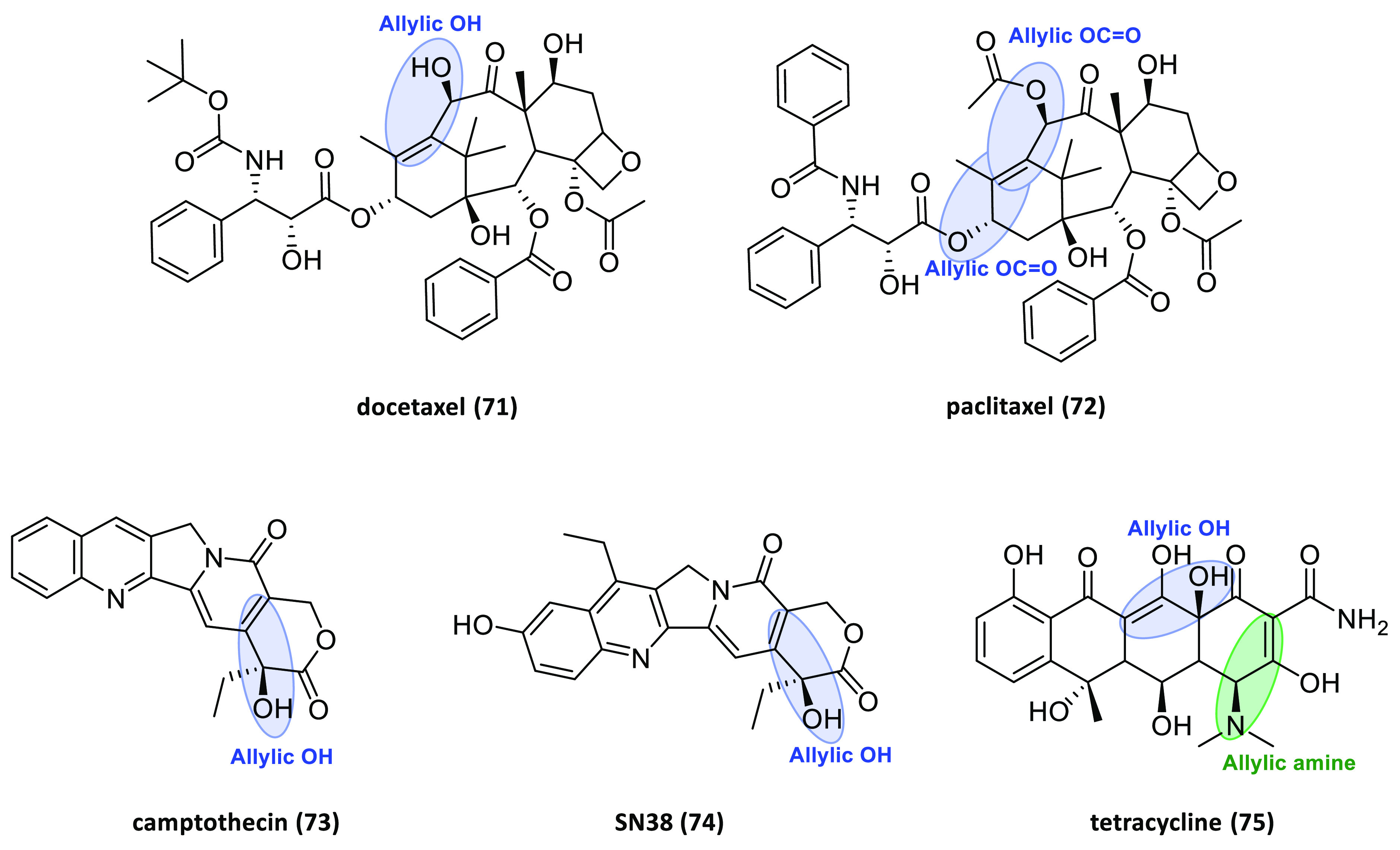
Structures of anticancer drugs, with allylic regions marked.

In the clinic, there are chemotherapy drugs that contain in their structure allylic regions (Figure 16). Docetaxel (**71**), an antineoplastic widely used in the clinic, incorporates a single allylic OH. However, paclitaxel (**72**), which is very similar to docetaxel, has an acetylated allylic OH, which will be deacetylated by cellular esterases. The other allyl ester presented in its structure could be hydrolyzed in the tumor, releasing a second allyl alcohol. Camptothecin (**73**), one of the most common anticancer drugs, has an allylic OH. Another of the camptothecin analogs used for cancer treatment is irinotecan, which is a prodrug that hydrolyzes to give the active compound SN38 (**74**). This active metabolite also has an allylic OH. Similarly, the tetracyclines (**75**) also exhibit in their structure an allylic OH and an allylic amine.^199^

When considering these drugs, it seems that the presence of heteroatoms, including O, N, or S, in the allylic position becomes relevant. These heteroatoms probably provide a relatively labile bond to the carbon, facilitating interactions with the target amino acids. Thus, the presence of the allylic region in the structure of anticancer drugs is not a limitation. It is a very small group, of which we have demonstrated anticancer activity in this work, and which can be introduced into the structure as part of very different chemical functions. In addition, it increases the lipophilic solubility of the molecule, improving its penetration through membranes

Finally, it is important to highlight the role that nanotechnology can play in advancing the use of chemotherapy. Conventional chemotherapy is based on small, non-selective molecules that do not differentiate between healthy and cancerous cells. These molecules are very active and, in many cases, succeed in curing or slowing the progression of cancer. The biggest problem they present is their high toxicity to healthy cells.

Therefore, nanomedicine offers the right tool to solve this problem. The encapsulation of chemotherapeutics in nanotechnological systems increases their bioavailability and concentration in tumor tissues, improving their pharmacokinetics and release profile and minimizing side effects. These advantages of nanotechnology are largely due to size and adjustable surface properties. The enhanced permeability and retention (EPR) effect is a concept that attempts to explain why molecules of certain sizes (such as nanoparticles) tend to accumulate more in tumor tissues than in normal tissues. The general explanation is based on specific defects in the tumor microenvironment, such as defects in lymphatic drainage, together with increased permeability of the tumor vasculature, to allow nanoparticles (<200 nm) to accumulate in the tumor microenvironment. On the other hand, by modulating the materials used for encapsulation and surface properties, controlled drug release is achieved through different events such as ultrasound, pH, heat, or material composition.

Lipid or protein systems that are biocompatible and biodegradable are used most commonly. In this Perspective, the use of nanotechnology has been mentioned on several occasions. One example is the formation of liposomes loaded with **5** and decorated on their surface with the RAGE antibody, which is overexpressed in triple-negative breast cancer cells, targeting them.^61^ Also, **49** (Figure 12) has been encapsulated in DOTA-PLGA nanoparticles decorated on their surface with HA for the treatment of colon cancer. HA recognizes the CD44 receptor that is overexpressed on tumor cell membranes.^178^ These are examples of how nanomedicine can be used, in these cases for targeted therapy, to get the drug to its target and prevent it from interacting in undesired areas, in order to reduce its toxicity. The microenvironment of tumor cells is very different from that of healthy cells, and these differences can be used to develop targeted therapies.^200,201^

Currently, there are nanopharmaceuticals approved by regulatory agencies for cancer treatment, such as Doxil (doxorubicin liposomes), Onivyde (irinotecan liposomes), Abraxane (paclitaxel protein conjugate), and Marqibo (vincristine liposomes). Therefore, nanomedicine is a viable strategy to reduce the potential hepatotoxicity of allylic derivative molecules during the development of new antitumor drugs.^202^

## 4 Summary and Perspectives

The development of more effective and less toxic molecules for the treatment of cancer is still a challenge to which the entire scientific community is committed. In this context, bioactive molecules derived from natural sources have a long history of use in the prevention and treatment of different diseases, especially in cancer, where they have played an essential role. This is the case of allyl derivatives, such as DAD, allicin, eugenol, and myristicin, present in garlic, cloves, nutmeg, and thyme. Similarly, allyl-containing molecules synthesized in the laboratory have exhibited effective anticancer activity *in vitro* and *in vivo*. In this Perspective, we have highlighted **38** and its derivative **39**, both containing an allyl chain, which are potent caspase activators and selectively induce apoptosis. Moreover, the *N*-allyl derivatives **49** and **51** have demonstrated potent *in vitro* and *in vivo* antitumoral activity, **49** reaching Phase III clinical trials. Therefore, the use of allylic fragments appears to be a useful tool in the design of new anticancer drugs.

From the point of view of bioactivity, in this Perspective, we have shown that the accumulated evidence indicates that allylic compounds exert their efficacy through multiple cellular pathways, although the caspase-dependent apoptosis is the main one. In addition to inducing apoptosis, some of the compounds are capable of inhibiting cell migration and invasion through up-regulation of miR-383-5p and inhibition of the ERBB4/PI3K/Akt pathway. This point is essential in the design of novel anticancer drugs, since these two characteristics of cancer are manifested in advanced stages and being able to develop effective drugs could mean an improvement in the cure rate of patients. Another key point is to develop molecules that act on specific therapeutic targets, in order to achieve better efficacy with fewer adverse effects. Thus, **5** down-regulates LIMK1, whose overexpression is associated with increased migration and invasion of colon cancer cells. Likewise, **6** has been reported to inhibit Trx reductase, which plays a key role in breast cancer metastasis, and to inhibit Srx, a new antioxidant enzyme that is overexpressed in a variety of cancers. Finally, not only have allyl derivatives been shown to decrease cell growth and proliferation, but they are also capable of arresting the cell cycle in G0/G1 and G2/M phases.

However, certain issues of some allyl derivatives must be taken into account, such as hepatotoxicity, genotoxicity, and carcinogenicity. The possible toxicity of these compounds is due to the electrophilic nature of the double bond or its epoxide, which alkylates nucleophilic biological components such as the purine bases of DNA or some amino acids. Among the allylic compounds studied in this Perspective, two major groups may be highlighted: (1) when the allylic chain is substituted with a sulfur atom, as exemplified by compounds in garlic, and (2) when the allylic chain is attached to a benzene ring, as exemplified by alkenyl benzenes. As we have seen earlier in this Perspective, garlic constituents can interact with biological proteins that present free thiols through reaction with the sulfur atom at the allylic position. Nevertheless, in alkenyl benzenes the allylic fragment plays a key role in interactions with biological proteins since the methylene group in these compounds is both benzylic and allylic, making this carbon more reactive.

In the clinic, there are drugs for the treatment of cancer that follow this mechanism of action, such as cyclophosphamide, chlorambucil, cisplatin, and oxaliplatin. However, these types of molecules do not differentiate between the DNA of healthy cells and the DNA of tumor cells, causing numerous side effects. On the other hand, the metabolism of these allylic derivatives can also generate intermediate metabolites with carcinogenic and mutagenic potential, such as epoxides and acrolein. As has been demonstrated, there are enzymes in the organism responsible for the elimination of epoxides, such as glutathione *S*-transferase and epoxide hydrolase. The first one forms conjugates with GSH that will later be eliminated in the urine. Epoxide hydrolase, which hydroxylates the epoxide, gives rise to more polar, non-harmful compounds, which are also typically eliminated in the urine. On the other hand, if the metabolism of the allylic derivatives results in acrolein, it is also conjugated with GSH to give mercapturic acid derivatives which are excreted in the urine.^191^ Therefore, although potentially harmful intermediates are formed, the human body has enzymatic mechanisms for their elimination. The presence of allylic regions in the structure does not mean that hepatotoxic metabolites will be formed during metabolism, since alcohol derivatives (safe) can also be generated, which are excreted in the urine.

**Figure 17:**
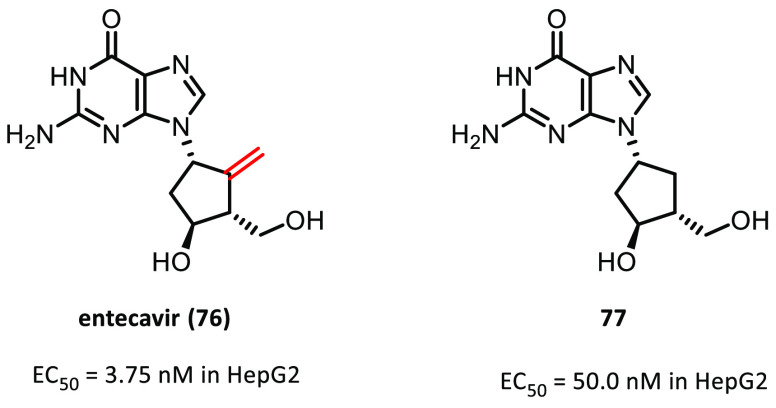
Structures of entecavir and its analog.

As we have shown in this Perspective, some molecules are designed as prodrugs without an overt allylic moiety in their structure. This is the case for **66** and **67**, which require bioreduction to generate the allylic element, and that is a critical aspect in exerting their alkylating action.^193,194,198^ This bioreduction is carried out in hypoxic cells, generally tumor cells, achieving greater selectivity against them. **67** showed good cytotoxic activity and a lack of toxicity in bone marrow cells. However, its short half-life hindered its systemic action. Therefore, it was tested locally against early-stage superficial bladder cancer, where it reached Phase III clinical trials.^196^ Additionally, there are other allylic molecules used for the treatment of different diseases, such as the antiviral entecavir (**76**, Figure 17). Entecavir (**76**) is a guanosine nucleoside analog that is used to treat liver infection caused by the hepatitis B virus. It is characterized by an exocyclic methylene group. The analog **77**, where this exocyclic double bond is absent, exhibits about 10-fold lower potency than **76** in HepG2 cells, demonstrating the importance of this double bond. Moreover, in HepG2 cells the CC_50_ was about 30 μM, which gives a selective index over 8000.^203^

There are also drugs used in the clinic which mask the allylic regions in their complex structure. Most of them have in common that the allylic region is part of carbocycles and is attached to a CH_2_ polar atom (N, O, or S) that polarizes the bond, facilitating interaction with other nucleophilic molecules. Finally, the use of nanomedicine offers great opportunities in the area of medicinal chemistry. Nanoencapsulation of chemotherapeutic drugs increases bioavailability and concentration in tumor tissues, minimizing adverse effects. In many cases, tumors express proteins on their surface that allow cancer cells to grow rapidly or abnormally. This situation can be used to design targeted therapies that act selectively on one type of tumor, as in TNBC, where RAGE ligand is overexpressed. This feature has been used to design nanoparticles loaded with DAD and decorated on their surface with RAGE, allowing them to be recognized by tumor cells that express its ligand.^61^ Similarly, **49** was encapsulated in nanoparticles decorated with HA for the treatment of colon cancer. HA is recognized by the CD44 receptor, which is overexpressed on tumor cell membranes.^178^ Both this nanoformulation and the previous one led to improved efficacy *in vitro* and *in vivo*. Therefore, the use of nanotechnology can reduce the limitations of allyl derivatives and achieve more effective therapies with fewer adverse effects.

Another key point during the design of novel therapeutic molecules is to consider bioavailability, since it will determine the amount of drug that reaches the circulatory system/target organ to exert its action. For this purpose, the concept of drug-likeness is used, and it is estimated from the chemical structure of the molecule. As the allyl fragment is small, its introduction into active molecules may not result in a significant change in solubility or molecular weight, and thus it is not expected to affect the molecule’s intestinal absorption and distribution. Moreover, allyl derivatives can undergo chemical changes during phase I of hepatic metabolism that give rise to more active and reactive molecules, such as epoxides. Alternatively, in phase II of hepatic metabolism, once their action has been exerted, they can be conjugated with proteins that facilitate their elimination. Hence, the introduction of the allylic motif in a molecule would favor its drug-likeness as well as providing high anticancer activity.

In addition to the above, an advantage of allyl fragments is that they can be easily chemically modified at the double bond site. For example, Diels–Alder cycloadditions can be carried out with conjugated dienes, giving rise to new derivatives. Moreover, some antineoplastic agents used in the treatment of cancer contain the allyl motif accompanied by nitrogen or oxygen in the form of alcohol (rapamycin), ether (amphotericin B), ester (paclitaxel), amine (vinblastine), or amide (manumycin A). Likewise, in other anticancer drugs the allylic moiety is generated after their metabolic oxidation, such as etoposide or gefitinib, being their metabolite active against cancer.^199,204,205^ Therefore, the presence of chemotherapeutic agents with the allyl motif currently in clinical use, along with all the literature collected in this Perspective, justifies the continued use of allyl fragments in the design of novel anticancer candidates

In summary, natural and synthetic molecules containing allyl fragments stand out as promising leads for the development of new anticancer drugs, showing preclinical efficacy in a large number of cancer models. Moreover, these effects were achieved through a wide variety of mechanisms of action, encompassing apoptosis, autophagy, Trx, and EMT. In some cases, toxicity issues were observed that can be solved through aforementioned strategies. Therefore, the development of new molecules containing allylic chains could lead to effective drug candidates for cancer treatment in the near future.

## Abbreviations Used

17-AAG: 17-allylamino-17-demethoxygeldanamycin
ADF: actin-depolymerization factor
AIF: apoptosis-inducing factor
AR: androgen receptor
BAX: Bcl-2 associated X protein
Bcl-2: B-cell lymphoma 2
CAF: cancer-associated fibroblasts
CDK2: cyclin-dependent kinase 2
CFW: Carworth Farms White
CCL2: C–C motif chemokine ligand 2
CSCs: cancer stem cells
CTR: calreticulin
CYP450: cytochrome P450
DAD: diallyl disulfide
DAS: diallyl sulfide
DAT: diallyl trisulfide
DHT: dihydrotestosterone
DJ-1: protein deglycase
EMT: epithelial–mesenchymal transition
EPR: permeability and retention
ER: endoplasmic reticulum
ER-α: estrogen receptor alpha
ERK1/2: extracellular signal-regulated kinase 1/2
FEMA: Flavor and Extract Manufacturers Association
GRAS: generally recognized as safe
GSH: glutathione
HCC: human hepatocellular carcinoma
HIF-1: hypoxia-inducible factor 1
IARC: International Agency for Research on Cancer
IGF-1: insulin-like growth factor-1
LIMK1: LIM kinase 1
MAO: monoamine oxidase
MAPK: mitogen-activated protein kinase
MDM2: mouse double minute 2
Mfn: mitofusin
miRNA: microRNA
MMP2: matrix metalloproteinase 2
MMP9: matrix metallopeptidase 9
MPST: mercaptopyruvate sulfurtransferase
NF-kB: nuclear factor kappa B
Nrf2: nuclear factor erythroid 2-related factor 2
NS: natural sources
NSAIDs: non-steroidal anti-inflammatory drugs
PAC-1: procaspase-activating compound 1
PAH: polycyclic aromatic hydrocarbons
PCNA: proliferating cell nuclear antigen
PD-L1: programmed death ligand 1
PEG: polyethylene glycol
PSA: prostate-specific antigen
Rac1: Rac Family Small GTPase 1
RAGE: receptor for advanced glycation end products
RhoGDPI2: RhoGDP dissociation inhibitor
SAC: S-allyl cysteine
SAMC: *S*-allyl-mercaptocysteine
Se: selenium
Srx: sulfiredoxin
STAT3: signal transducer and activator of transcription 3
TGF-β1: transforming growth factor-beta 1
Timp-3: tissue inhibitor of metallopeptidase inhibitor 3
TNBC: triple-negative breast cancer
Trx: thioredoxin
